# Disparities in access to and outcomes of minimally invasive surgery: a scoping review

**DOI:** 10.1007/s00464-026-12768-8

**Published:** 2026-04-02

**Authors:** Shaneeta Johnson, Zahra A. Fazal, Kelsie Campbell, Neera Patel, Elizabeth Wall-Wieler, Ana Yankovsky, Loretta Erhunmwunsee

**Affiliations:** 1 Department of Surgery, Meharry School of Medicine, Meharry Medical College, Nashville, TN USA; 2 Department of Population Health, Meharry School of Global Health, Meharry Medical College, Nashville, TN USA; 3 Intuitive Surgical, Sunnyvale, CA USA; 4 Department of Surgery and Department of Population Science, City of Hope Medical Center, 1500 East Duarte Road, Duarte, CA 910101 USA

**Keywords:** Minimally invasive surgery, Disparities, Surgery, Social determinants of health, Robotics, Surgical access

## Abstract

**Background:**

Minimally invasive surgery (MIS) has been associated with improved clinical outcomes compared to open surgery for various procedures. However, disparities in access to MIS persist across race/ethnicity, sex, geography, and payor status. This scoping review aims to synthesize existing evidence on disparities in MIS use and outcomes and to highlight key gaps in the literature.

**Methods:**

A comprehensive literature search was conducted in PubMed and Scopus from 1 January 2020, to 18 October 2024, for US peer-reviewed research on access to and outcomes of MIS in equity-seeking groups. Title/abstracts and full text were independently reviewed, with conflicts resolved by consensus. Information on study characteristics and both clinical and non-clinical outcomes were extracted. Factors associated with adverse clinical outcomes or a lower likelihood of access to MIS were grouped as themes and represented by surgical specialty using a bubble plot. A protocol for this review was pre-registered on Open Science Framework and updated through the course of the study.

**Results:**

A total of 88 articles involving 11,647,821 patients were included in the review. The most frequently reported domain of disparity was race/ethnicity (*n* = 62 papers), and the most frequently analyzed specialty was general surgery (*n* = 41 papers). Overall, a decrease in likelihood of MIS was reported among patients who were Black, Hispanic or Native American, uninsured or on Medicaid, from lower socioeconomic status (SES), and those in rural locations. Adverse clinical outcomes of MIS, such as readmissions and complications, were associated with patients who were Black, female, of lower SES, and on Medicaid. Key gaps in the literature were noted in the investigation of disparities across geography (rurality/urbanicity), language and at the intersection of multiple domains of disparity.

**Conclusion:**

Our review provides important considerations for understanding the inequities across patient groups in access to and outcomes of MIS.

**Graphical abstract:**

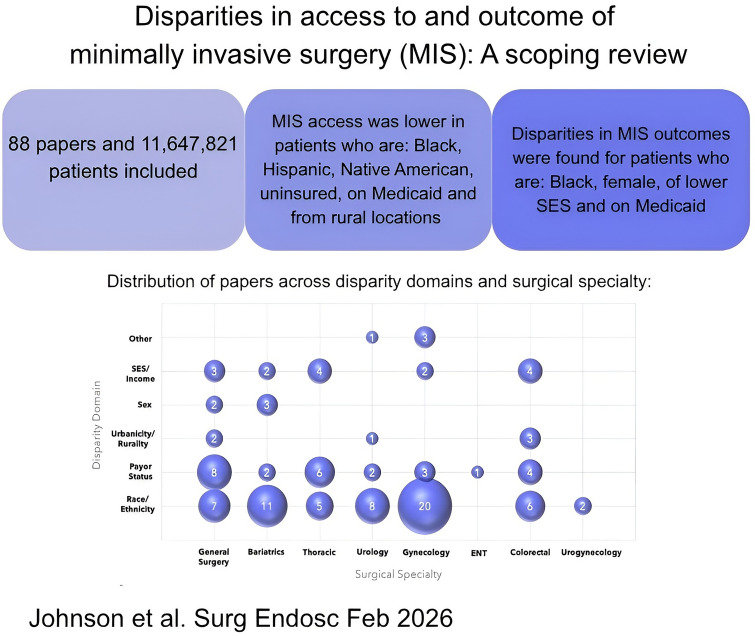

**Supplementary Information:**

The online version contains supplementary material available at 10.1007/s00464-026-12768-8.

Minimally invasive surgery (MIS), including laparoscopic and robotic-assisted surgery (RAS), has gained increased adoption over the past decade [1]. MIS has been shown to improve clinical outcomes, such as shorter length of stay and lower rates of perioperative complications, across various specialties compared with open surgery [2–5]. However, the benefits of MIS are not experienced equally among all groups, due to differences in access, including the impact of MIS deserts [6].

A foundational systematic literature review by de Jager et al. found that surgical quality metrics, such as surgical delay, readmission, complications, and the use of minimally invasive procedures, varied across several equity domains [7]. Disparities in MIS access and surgical outcomes exist based on a myriad of factors, including sociodemographic measures, such as race, ethnicity, payor status, socioeconomic status (SES), and geographical residential area [8–11]. Additionally, there has been a plethora of studies that report similar findings related to MIS disparities specific to surgical specialties, including Urology, General Surgery, Gynecology, Thoracic Surgery, and Surgical Oncology [12–18].

Current literature exploring inequities in MIS typically focuses on specific populations or procedures, thereby limiting a comprehensive assessment of disparities across the broader MIS landscape. The extensive scope of outcomes, disparity domains, and surgical specialties present in the literature underscores the need for a holistic scoping review to summarize these results.

The objective of this review is to synthesize existing research on disparities in MIS access and outcomes, with a focus on critical drivers of health, particularly race/ethnicity, sex, urbanicity, SES, and payor status. This review aims to enhance understanding of equity in minimally invasive surgical care by identifying trends and gaps in the literature, thus supporting efforts to promote inclusive healthcare policies. Addressing disparities in MIS is essential to ensuring that all patients, regardless of background, benefit from advancements in surgical technology and improved health outcomes.

Specifically, the research questions guiding this scoping review are as follows:

(i) What patient populations are identified in the current literature that experience inequities in accessing minimally invasive surgeries?
(ii) What outcomes differ across these identified patient populations when undergoing MIS?

## Methods

### Protocol registration

This review was prospectively registered in Open Science Framework (OSF) on 24th October 2024, and updated on 5th September 2025 (10.17605/OSF.IO/DH4P3) [19]. The scoping review was conducted following the Preferred Reporting Items for Systematic Reviews and Meta‐Analyses extension for Scoping Reviews to provide transparency and reproducibility [20].

### Search strategy

Literature indexed in PubMed and Scopus was examined using MeSH terms broadly combining equity and MIS (Supplemental File 1) from 1 January 2020 to 18 October 2024. These databases were chosen for their extensive coverage of surgical and outcome disparities literature, ensuring a comprehensive search that balances study scope and resource constraints. EndNote 20 (Clarivate, Philadelphia, PA, USA, London, UK) was used for deduplication of studies, after which studies meeting the inclusion and exclusion criteria were retained. The eligibility criteria allowed for meeting the research objective while minimizing resource constraints. Gray literature was excluded, given the large volume of peer-reviewed literature published on this topic. The full search strategy is included in Appendix (Supplemental File 1).

Inclusion criteria were as follows:

(i) Original peer-reviewed literature.
(ii) Published between 1 January 2020 and 28 October 2024.
(iii) The primary aim of the article being either access to or outcomes of MIS in comparison to a reference group. For the purposes of this review, the definition of minimally invasive surgery was restricted to include only soft-tissue surgery that is either laparoscopic (including Video-Assisted Thoracoscopic Surgery) or Robotic-Assisted Surgery (RAS).
(iv) A focus on one or more domains of sociodemographic inequity.

Exclusion criteria were as follows:

(i) Studies that were not in English.
(ii) Clinical safety studies and non-inferiority studies.
(iii) Studies that did not focus on adult patients (< 18 years of age, physician-based studies).
(iv) All forms of Gray literature (e.g., conference abstracts, editorials, etc.).

### Screening and full-text review

Title and abstract screening were conducted by author ZAF and then verified separately by author KC following the inclusion and exclusion criteria. If an index was missing an abstract, it was automatically moved to full-text for comprehensive review. Every reference in the full-text stage was screened independently by the same two authors. Inter-author consensus rates were 99.6% and 90.9% for each stage, respectively. Differences were adjudicated through collaborative review during weekly team meetings. Laser AI (Evidence Prime, 2024) was used as an evidence synthesis software for organization and screening. For each stage, 20 articles were randomly sampled from the records and pilot-tested to ensure consistency between authors in the application of the inclusion/exclusion criteria. Detailed screening instructions for the title and abstract stage, the full-text stage, as well as pilot test results, can be found in the OSF protocol.

### Data extraction and analysis

The following information was extracted from articles that met the eligibility criteria by author ZAF and validated separately by author KC: last name of the first author, year of publication, study design, country of focus, surgical specialty (as defined by the American College of Surgeons) [21], surgical procedure, sample size, domain of disparity, operationalization of disparity, outcome of interest, and key findings. These items included in the data charting were iterated during the data extraction pilot testing and agreed upon by all coauthors as relevant to the study aim. Detailed instructions for the extraction, results from the pilot test, and justifications for amendments can be found in the OSF protocol. A PRISMA flow diagram was used to track the study selection methods.

From the extracted data, the same two authors independently assessed the codes that emerged across the included studies. These codes were then grouped by disparity domain and surgical specialty after consensus discussion. Evidence was then summarized into tables and visual outputs. Given that this review is scoping and foundational in nature, a formal bias assessment was not deemed necessary to meet the study objectives and was therefore not performed for the included studies.

## Results

**Fig. 1:**
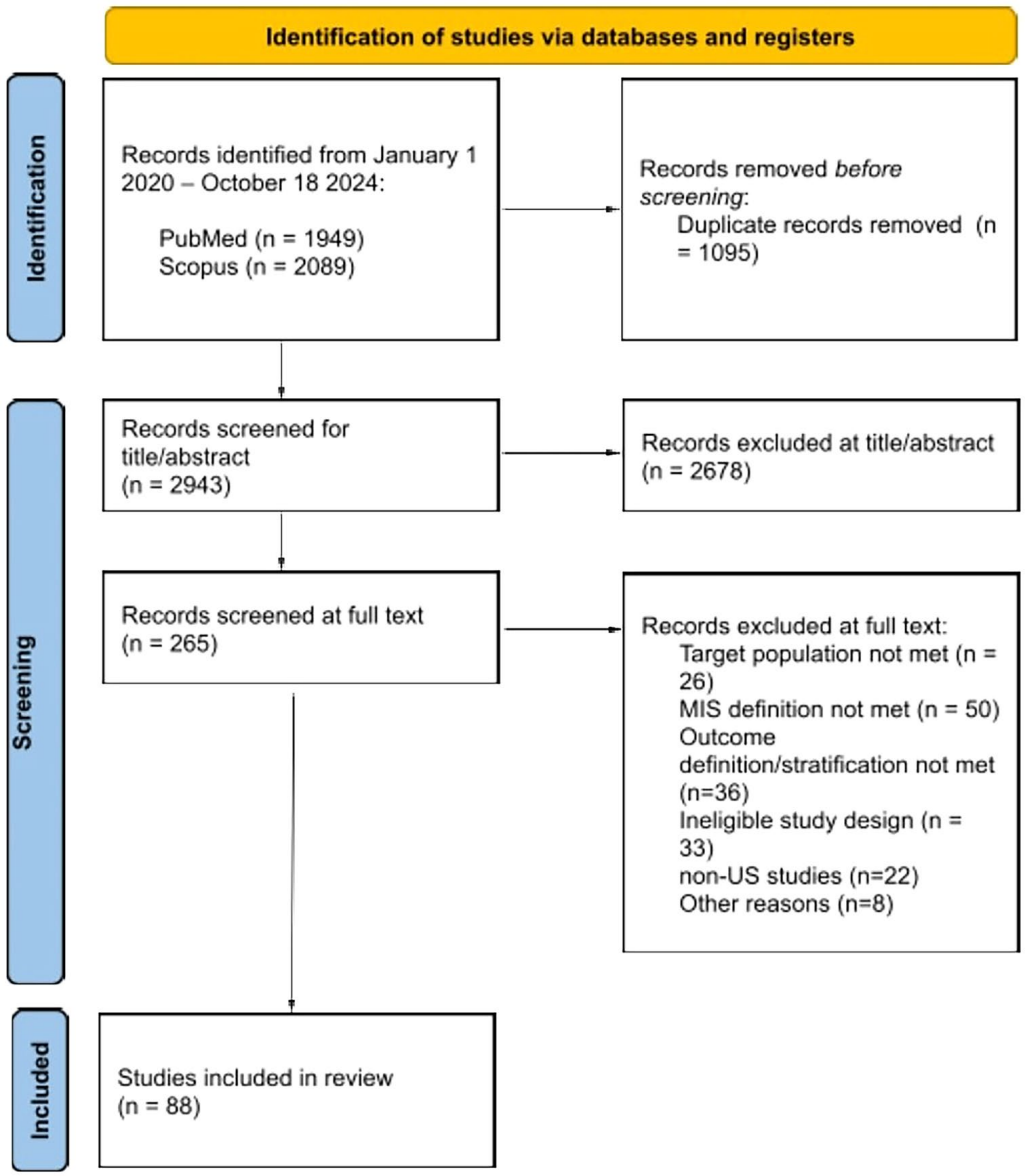
PRISMA Search selection process. *MIS* minimally invasive surgery, *US* United States

The initial search resulted in 4038 records from PubMed (*N* = 1949) and Scopus (*N* = 2089). Duplicates (*N* = 1095) were removed, and 2943 studies were eligible for title and abstract screening. After removing 2678 studies that did not meet our criteria, we retrieved the full text of the remaining 265 studies, of which 88 were included in the final review. Notably, we removed 22 studies conducted outside the United States (US) due to heterogeneity in definitions of disparity domains, such as race and payor status, across countries. The PRISMA flow diagram (Fig. 1) shows the study selection.

### Characteristics of included studies

**Fig. 2:**
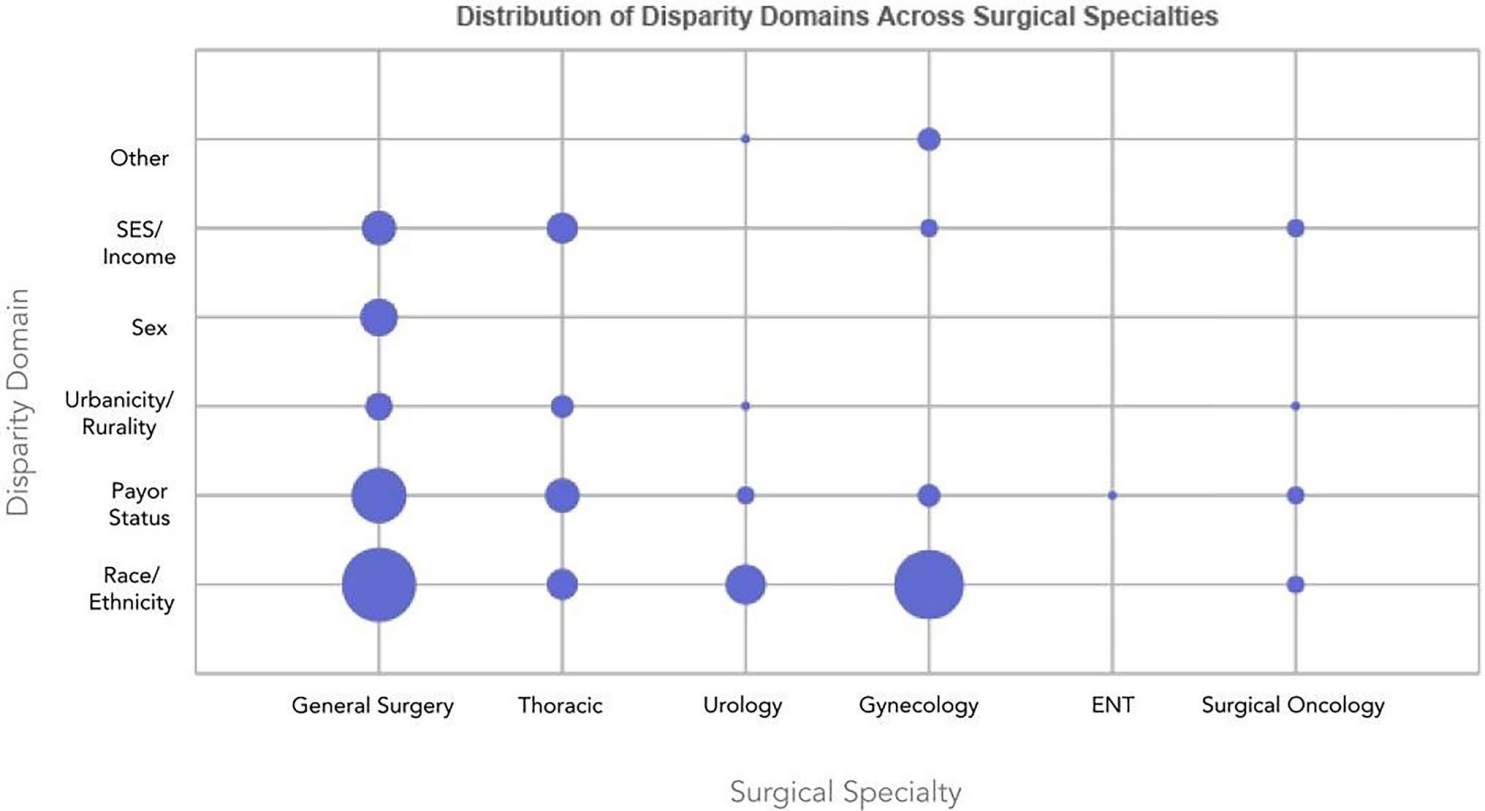
Bubble plot showing frequency of disparity domains by surgical specialty. *SES* socioeconomic status, *ENT* ear nose and throat

The 88 studies included in the review comprised a total of 11,647,821 patients across all surgical approaches; most were retrospective cohort studies (*n* = 79), followed by cross-sectional (*n* = 6), prospective cohort (*n* = 2), and 1 case–control study. Surgical specialties represented in the studies included: general surgery (*n* = 41), gynecology (*n* = 24), cardiothoracic (*n* = 11), urology (*n* = 9), surgical oncology (*n* = 2), and ENT (*n* = 1). Studies assessed either a single domain of inequity (*n* = 66) or combined several domains (*n* = 19), but only three studies adopted a truly intersectional approach, analyzing race/ethnicity with either urbanicity, sex, or income. All studies adjusted for at least one potential confounder. Findings by theme and surgical specialty are summarized using a bubble plot (Fig. 2).

### Themes from included studies

The major themes from the included studies on disparities in access to MIS and/or the outcomes of MIS were as follows: (i) race/ethnicity, (ii) sex, (iii) urbanicity/rurality, (iv) payor/insurance status, (v) SES/income, and (vi) others. The findings from each theme are summarized below.

#### Race and ethnicity

Sixty-two studies (*n* = 7,713,746 patients across all surgical approaches) assessed racial/ethnic disparities in MIS. Of these, 3 studies were intersectional [18, 22, 23], and 16 studies explored multiple domains [24–38]. Additionally, 39 studies reported MIS access disparities compared to open surgery [18, 22, 25–34, 36–61], 17 studies reported disparities in clinical outcomes [23, 35, 47, 62–75], and 6 studies explored both [12, 24, 76–79]. The most consistent disparity in access to MIS was observed among Black patients, who had lower odds of undergoing MIS across multiple procedures as reported by five studies [35, 45, 62, 63, 67]. This included the lowest reported odds for hysterectomy (aOR 0.33, CI 0.21 to 0.51) [45]. Furthermore, in six studies across multiple procedures and within the specialties of bariatrics and gynecology, Hispanic patients had reduced odds of MIS [35, 39, 45, 63, 67, 73], with the lowest observed odds for MIS hysterectomy (aOR 0.35, 95% CI 0.20 to 0.60) [45]. Native Hawaiian/Pacific Islander (NHPI) women also demonstrated markedly reduced likelihood of undergoing MIS hysterectomy (OR 0.31, 95% CI 0.10 to 0.56) [45] and were less likely to receive laparoscopic sacrocolpopexy (OR 0.68, CI 0.58 to 0.79) [50]. In contrast, some Asian subgroups had higher MIS access among procedures, such as colorectal surgeries [43] and thoracic lobectomy [28].

Several studies reported no significant differences by race/ethnicity for certain settings or procedures. For example, these studies reported a similar likelihood of laparoscopic colectomy in Hispanic vs. White patients [57], no difference in planned MIS hysterectomy by race within a single health system [52], and no association of approach with ethnicity in radical cystectomy [80]. Furthermore, structural factors also contributed to disparities in radical prostatectomy, as Black and Hispanic patients were more likely to be treated by low-volume robotic surgeons or at hospitals without robotic capacity, substantially reducing their likelihood of receiving MIS [47].

A total of 17 studies reported clinical outcomes of MIS. Across racial and ethnic groups, Black patients had the greatest overall risk of adverse outcomes, with higher odds of postoperative complications [23, 62, 67, 71], readmissions [62, 63, 65, 67], and re-interventions [62] compared with White patients. Black patients also had longer operative times, longer lengths of stay, and higher morbidity in bariatric cohorts [65, 67]. For sleeve gastrectomy (SG) and gastric bypass, Black patients of both sexes had significantly higher complication risks [23]. Furthermore, for Roux-en-Y gastric bypass (RYGB), they had increased odds of renal-related readmissions [63] and pulmonary embolism [67]. Hispanic patients also demonstrated elevated risks for select outcomes, including venous thromboembolism-related readmissions after RYGB, leak-related readmissions after SG [63], and—on sub-analysis of laparoscopic colorectal resections—higher odds of major complications [71]. Interestingly, one study reported that Black and Hispanic patients saw larger absolute reductions in major events [Relative Excess Risk Due to Interaction (RERI): − 0.32, CI − 0.71 to − 0.03; RERI: − 0.27, CI − 0.77 to 0.09, respectively] and prolonged length of stay (RERI: − 0.63, CI − 0.98 to − 0.35; RERI: − 0.93, CI − 1.46 to − 0.51, respectively) with robotic-assisted radical prostatectomy versus open radical prostatectomy than White patients [47].

Conversely, eight studies reported some conflicting evidence to the overall trends in outcomes observed. Five studies reported no racial/ethnic variation in outcomes, including postoperative pain and analgesia for hysterectomy [68], myomectomy blood loss [69], medication refill requests for prostatectomy [70], complications and mortality after partial nephrectomy [66, 72], and survival after partial nephrectomy [72]. Additionally, Black patients showed lower odds of leak- and cardiovascular-related readmissions than White patients for RYGB [63], and Hispanic patients had lower major and minor complication rates after RYGB despite higher leak rates, with no overall difference for SG [73]. Finally, in a matched bariatric cohort for procedures RYGB and SG, White patients were more likely to experience bleeding and superficial surgical-site infection when compared to Black patients [64].

Evidence for American Indian or Alaska Native patients was extremely limited, with only one study reporting higher odds of re-intervention for RYGB and SG [62]. Several groups, including Asian, Native Hawaiian/Other Pacific Islander, and multiracial subgroups, were also sparsely represented. Occasionally, “other/unknown” categories showed disparities (e.g., higher odds of laparotomy or lower-MIS use) [42, 45], yet these findings were too heterogeneous to interpret, resulting in limited insights. Notable findings and trends related to urbanicity as a domain are summarized (Table 1). See Supplemental Table 2 for all studies included within this domain (Supplemental File 2).

**Table 1:** Characteristics of the 62 included studies on race/ethnicity

Characteristic	Number (*N* = 62)	Percentage (%)
**Study type**		
Retrospective cohort	55	88.7
Prospective cohort	1	1.6
Cross sectional	5	8.1
Case–control	1	1.6
**Surgical specialty**		
Gynecology	22	35.5
Urology	8	12.9
General surgery	25	40.3
Cardiothoracic	5	8.1
Surgical oncology	2	3.2
**Outcome reported**		
Clinical	17	27.4
Non-clinical (access-related)	39	62.9
Both	6	9.7

#### Sex

Seven studies (*n* = 2,133,671 patients across all surgical approaches) assessed sex disparities in the MIS space, of which one was intersectional [23], and three studies explored multiple domains [27, 35, 46]. Access findings were procedure-specific and bidirectional: women were less likely to receive minimally invasive endoscopic groin hernia repair (30% vs. 60% in men) [81] or robotic proctectomy [45], but more likely to undergo laparoscopic or robotic ventral or incisional hernia repair (aOR 1.26, 95% CI 1.10 to 1.44) [27, 82].

Regarding clinical outcomes, male sex was associated with increased complications and mortality after RYGB and SG [83]. In contrast, an intersectional analysis across duodenal switch (DS), SG, and RYGB found no overall sex differences in postoperative complications, with the exception of higher complications among non-Hispanic White males vs. females after SG [23]. Notable findings and trends related to urbanicity as a domain are summarized (Table 2). See Supplemental Table 3 for all studies in this domain (Supplemental File 2).

**Table 2:** Characteristics of the seven included studies on sex

Characteristic	Number (*N* = 7)	Percentage (%)
**Study type**		
Retrospective cohort	7	100
**Procedure area**		
General surgery	7	100
**Outcome reported**		
Clinical	3	42.9
Non-clinical (access-related)	4	57.1

#### Urbanicity/rurality

Nine studies (*n* = 1,344,801 patients across all surgical approaches) assessed geographic disparities in access to MIS, of which six assessed it as a secondary focus and two as an intersectional focus. Only one study comparing rural and urban hospitals assessed geographic disparities as the primary focus and found no difference in access to MIS for cholecystectomy (aOR 1.93, 95% CI 0.73 to 5.13) [84].

Among the studies that compared receipt of MIS by urbanicity as a secondary objective, two found that residents of rural areas were significantly less likely to undergo MIS for colorectal surgery (aOR 0.75, 95% CI 0.70 to 0.80) [26, 27]. Similar findings were highlighted for lobectomy when rurality was analyzed independently (aOR 0.87, 95% CI 0.70 to 0.91) [28], and also when it was analyzed with socioeconomic status and payor status [17]. An intersectional analysis of race and rurality found that rural White patients have lower odds for MIS compared to Black urban patients, suggesting that geography may exert an additive impact on access to MIS [18].

No studies assessed geographic differences in the clinical outcomes of MIS by urbanicity. Notable findings and trends related to urbanicity as a domain are summarized (Table 3). See Supplemental Table 4 for all studies included within this domain (Supplemental File 2).

**Table 3:** Characteristics of the nine included studies on urbanicity/rurality

Characteristic	Count (*N* = 9)	Percentage of category (%)
**Study type**		
Retrospective cohort	8	88.9
Cross sectional study	1	11.1
**Surgical specialty**		
General surgery	4	44.4
Cardiothoracic	3	33.3
Urology	1	11.1
Surgical oncology	1	11.1
**Outcome reported**		
Non-clinical (access related)	9	100
Clinical	0	0

#### Payor/insurance status

Twenty-eight studies (*n* = 3,845,003 patients across all surgical approaches) assessed payor status disparities in the MIS space, of which 12 studies analyzed it as a primary focus, 15 studies included it among multiple dimensions of disparity, and 1 study assessed it at an intersection with another domain. Of these studies, only two reported disparities in outcomes, while the remaining reported on access disparities, and one paper reported both.

Overall, studies found that Medicaid and uninsured patients had lower access to MIS than privately insured patients across colorectal surgery [22, 46, 85–88], hysterectomy [25], and lobectomy [17, 28, 34]. The disparity between MIS utilization by insurance status was as significant as 50% for some procedures like pancreatectomy (aOR 0.5, 95% CI 0.4 to 0.7]) [30].

Additionally, three studies examined the effect of Medicaid expansion, and two found that it was associated with an increase in MIS utilization across various procedures, including lung resection and surgeries for diverticulitis [89, 90]. One study, however, found no effect on MIS esophagectomy utilization due to expansion [91].

Outcome disparities were reported for (i) weight loss after bariatric surgery—equivalent for Medicaid patients [92, 93] and (ii) cost of surgery—higher in Medicaid patients, with one study reporting it to be as high as $5000 or more [86]. However, robotic-assisted surgery had a positive mitigating effect on the cost disparity between Medicare and privately insured patients, specifically for lung resection [94]. Finally, there was mixed evidence around access to MIS for Medicare patients, with some studies showing no disparity. In contrast, others showed a lower odds of access when compared to privately insured or Medicaid patients [30, 31, 36]. Findings and trends related to payor status as a domain are summarized (Table 4). See Supplemental Table 5 for all studies included within this domain  (Supplemental File 2).

**Table 4:** Characteristics of the 26 included studies on payor/insurance status

Characteristic	Count (*N* = 28)	Percentage of category (%)
**Study type**		
Retrospective cohort	25	89.3
Cross sectional study	3	10.7
**Surgical specialty**		
General surgery	15	53.6
Cardiothoracic	5	17.9
Gynecology	3	10.7
Urology	2	7.1
Surgical oncology	2	7.1
ENT (Ear, Nose and Throat)	1	3.6
**Outcome reported**		
Non-clinical (access related)	25	89.3
Clinical	2	7.1
Both	1	3.6

#### Socioeconomic status (SES)/income

Fifteen studies (*n* = 914,167 patients across all surgical approaches) assessed SES disparities, of which 3 studies had it as their primary focus, 10 studies included it among multiple dimensions of disparity, and 2 studies assessed it intersectionally. Of these studies, only two studies reported disparities in outcomes [95, 96], while the remaining studies focused on access disparities.

Overall, patients with lower SES were seen to have lower odds for receiving MIS compared to open surgery and were associated with worse outcomes for weight recurrence after bariatric surgery [96] and nearly double the odds of postoperative complications for pulmonary lobectomy (aOR 1.98, 95% CI 1.03 to 3.78) [95]. Of note, SES was operationalized in different ways across studies; the most frequent was ZIP-level data, such as distressed community indexes [96, 97], ZIP-based median income [17, 95, 97–99], and education-based proxies [98]. Notable findings and trends related to urbanicity as a domain are summarized (Table 5). See Supplemental Table 6 for all studies included within this domain (Supplemental File 2).

**Table 5:** Characteristics of the 15 included studies on socioeconomic status/income

Characteristic	Count (*N* = 15)	Percentage of category (%)
**Study type**		
Retrospective cohort	13	86.7
Cross sectional study	2	13.3
**Surgical specialty**		
General surgery	6	40.0
Cardiothoracic	5	33.3
Gynecology	2	13.3
Surgical oncology	2	13.3
**Outcome reported**		
Non-clinical (access related)	13	86.7
Clinical	2	13.3

#### Other

Four studies examined disparity domains outside those detailed above, primarily in hysterectomy cohorts. *Language:* one study found there was no difference in the use of a minimally invasive approach for cholecystectomy based on the primary language spoken (English: 99.8%, Non-English: 99.9%) [100], while another found that patients with a non-English primary language were significantly less likely to undergo minimally invasive hysterectomy compared to English-speaking patients (OR 0.60, CI 0.56 to 0.64) (Pena et al., 2024). *Distance and specialist access:* among patients living in counties without a gynecologic oncologist, Black patients who did not travel had markedly lower odds of MIS than non-Black patients who traveled (aOR 0.57, 95% CI 0.49 to 0.65; interaction aOR 0.60, 95% CI 0.40 to 0.90) [25]. *Hospital MIS volume:* care at centers where > 80% of hysterectomies were performed minimally invasively conferred nearly sixfold higher odds of MIS (aOR 5.89, 95% CI 4.51 to 7.68), yet non-Hispanic Black patients were disproportionately treated at lower-MIS hospitals [38]. *Hospital type:* robotic ureteral reimplantation cases were clustered among privately insured patients and at private for-profit institutions [101].

## Discussion

This is the first scoping review to summarize the existing literature on disparities in access to and outcomes of MIS in the US across multiple domains and surgical specialties. The comprehensiveness of this review, spanning 88 studies under strict inclusion criteria, allowed for the identification of key patient populations for whom disparities have been documented while also identifying gaps in the literature. Disparities were most frequently observed across race/ethnicity and payor status, with some evidence also documenting inequities by urbanicity, sex, and SES. Outcome disparities were observed for postoperative complications, readmissions, mortality, and cost of surgery. The literature on disparities was concentrated in general surgery, followed by gynecology and cardiothoracic surgery, with fewer studies in urology, ENT, and surgical oncology. The results of this scoping review demonstrate the evolving understanding of social determinants of health (SDOH) and underscore their role in shaping surgical care delivery.

Several disparity domains remain underexplored in the MIS literature. Only a few studies have evaluated the role of urbanicity or rurality, despite emerging research on “MIS deserts” suggesting that geographic disparities may intersect with other factors, such as income, insurance status, and the availability of MIS-trained surgeons [6]. As noted in our findings, residents of rural areas were significantly less likely to undergo MIS for colorectal and lobectomy procedures, and rural White patients had lower odds of MIS compared with Black urban patients. These findings suggest that geographic barriers, such as the centralization of complex MIS procedures at high-volume academic or expert centers concentrated in urban areas, may substantially limit access to MIS and, in some cases, exert a stronger influence on receipt of MIS than race. This underscores the need to consider how geographic and racial inequities intersect.

Only two studies in this review examined language as a primary disparity domain [100, 102], highlighting, as Joo et al. (2023) noted in their systematic literature review on the association of language barriers with perioperative and surgical outcomes, the scarcity of research on how language barriers contribute to perioperative health disparities [103]. Expanding research in these underrepresented areas and integrating multidimensional analyses that consider the intersection of race/ethnicity, language, geography, SES, and other SDOH could provide a more comprehensive understanding of inequities in MIS access and outcomes.

Despite race/ethnicity being the most frequently documented disparity domain, few studies reported how this construct was measured, and authors rarely distinguished between race and ethnicity, often adopting an ethno-racial construct without explicit justification. The way race and ethnicity are defined and categorized can meaningfully affect study findings, and prior research highlights the need for intentionality and transparency in epidemiologic studies [104]. We also observed frequent aggregation of diverse racial and ethnic groups—such as Native American, Pacific Islander, Middle Eastern, North African, and Asian—into a single “Other” category, which obscures group-specific patterns and risks overlooking meaningful disparities. More granular classification, justification of analytic decisions, and reporting of data collection methods can enhance the interpretability and equity relevance of research.

This scoping review has several important limitations. Many studies varied in how they defined MIS, and several did not specify which surgical approaches were included (e.g., their methods sections or Supplementary Files did not clarify which modalities were considered within the researchers’ definition of MIS). Robotics-assisted surgery and conventional laparoscopy share core characteristics (i.e., using specialized instruments and camera-guided techniques through small incisions to minimize tissue disruption) that have led to improved perioperative outcomes compared with open surgery [105]; however, they also differ across domains such as cost, length of stay, conversion rates, and other outcomes [106]. We therefore interpreted study findings cautiously, considering how each study defined MIS. Studies that did not clearly define MIS were therefore excluded due to difficulties in interpretation and comparison of findings across studies.

This review only searched two databases and did not critically appraise the quality of the evidence that would facilitate further stratification of studies. Additionally, it excluded Gray literature that might have captured emerging studies, as well as qualitative evidence within the MIS health equity area. Publication bias may also have influenced the results, with studies being more likely to be published if significant disparities are identified. Finally, although our review was comprehensive, we opted not to include global evidence, as disparity domains are conceptualized and measured heterogeneously across countries, thereby limiting comparability. Nonetheless, there is a critical need for more global research. Therefore, we intend to run the same search of non-U.S. studies that meet our inclusion criteria in a follow-up paper.

Despite these limitations, the review has considerable strengths, including its novel stratification of findings by domains of disparity as well as surgical specialties. By including studies across five domains of disparity and six surgical specialties, this review provides the first consolidation of literature on disparities in the MIS space, mapping both overrepresentation and underrepresentation of themes to guide future research and targeted interventions. Additionally, the review maintained rigorous reproducibility practices, including registering the protocol (including its amendment), recording consensus rates between the two independent screeners at each stage, and reporting findings in accordance with PRISMA-ScR guidelines. Finally, the inclusion of visuals from the PRISMA diagram, evidence tables, and bubble plot allows the findings from the study to be accessible to a wide range of stakeholders and decision-makers.

Given the important implications of interpreting disparities, future research on MIS and health equity would benefit from greater methodological clarity and consistency in both MIS definitions and the operationalization of SDOH variables. Identifying which groups and factors are linked to decreased MIS access and poorer outcomes post-MIS is critically important as we aim to improve outcomes for communities. The data within this review support efforts to design interventions and advocacy goals that champion equitable access, which is becoming increasingly important as institutional resources are further restricted, and insurance coverage for at-risk groups is decreased by recent legislative action. Without continued focus and action on such data, outcomes for already vulnerable groups will undoubtedly worsen.

Some studies that would otherwise have been relevant to our review were excluded because they combined multiple SDOH measures into a single composite predictor. While such approaches may be useful in specific contexts, they limit the ability to differentiate and quantify the unique contributions of specific factors, such as income, insurance status, or level of education. Future research should consider analyzing unique SDOH variables separately to improve identification of the most salient drivers of inequities in MIS access and outcomes.

## Conclusion

This scoping review summarized the evidence regarding disparities in minimally invasive surgery. The synthesis revealed that access to MIS was the lowest among patient groups who were Black, uninsured or insured with Medicare (compared to private insurance), female, and from rural communities. Clinical outcomes after MIS also varied, with higher complication rates, readmissions, and other adverse outcomes observed in similar patient groups.

Gaps in the literature regarding MIS disparities were found in urology and ENT specialties, as well as across language, urbanicity, and SES domains. This review provides foundational evidence of disparities across multiple domains and highlights research gaps that can be addressed to advance equitable patient care in surgical delivery.

## Supplementary Information

Below is the link to the electronic supplementary material.

Supplementary file1 (DOCX 72 kb)

Supplementary file2 (DOCX 1699 kb)

## References

[1] Bonner SN, Thumma JR, Dimick JB, Sheetz KH (2023) Trends in use of robotic surgery for privately insured patients and Medicare fee-for-service beneficiaries. JAMA Netw Open 6:e2315052. 10.1001/jamanetworkopen.2023.15052 37223903 10.1001/jamanetworkopen.2023.15052 PMC10209745

[2] Fan CJ, Chien H-L, Weiss MJ, He J, Wolfgang CL, Cameron JL, Pawlik TM, Makary MA (2018) Minimally invasive versus open surgery in the Medicare population: a comparison of post-operative and economic outcomes. Surg Endosc 32:3874–3880. 10.1007/s00464-018-6126-z 29484556 10.1007/s00464-018-6126-z

[3] Bogani G, Rossetti D, Ditto A, Martinelli F, Chiappa V, Leone C, Leone Roberti Maggiore U, Lorusso D, Raspagliesi F (2019) Minimally invasive surgery improves short-term outcomes of nerve-sparing radical hysterectomy in patients with cervical cancer: a propensity-matched analysis with open abdominal surgery. J Gynecol Oncol 30:e27. 10.3802/jgo.2019.30.e27 30740958 10.3802/jgo.2019.30.e27 PMC6393638

[4] Hajirawala LN, Krishnan V, Leonardi C, Bevier-Rawls ER, Orangio GR, Davis KG, Klinger AL, Barton JS (2022) Minimally invasive surgery is associated with improved outcomes following urgent inpatient colectomy. J Soc Laparosc Robot Surg 26:e2021.00075. 10.4293/JSLS.2021.00075 10.4293/JSLS.2021.00075 PMC8896814 35281708

[5] Hu JC, Wang Q, Pashos CL, Lipsitz SR, Keating NL (2008) Utilization and outcomes of minimally invasive radical prostatectomy. JCO 26:2278–2284. 10.1200/JCO.2007.13.4528 10.1200/JCO.2007.13.4528 18467718

[6] Mitzman B, Johnson S, Lichtveld M, Culbertson R, Fong ZV (2025) Minimally invasive surgery deserts: is there a role for robotic assisted surgery? J Soc Laparosc Robot Surg 28:e2024.00039. 10.4293/JSLS.2024.00039 10.4293/JSLS.2024.00039 PMC11723336 39801727

[7] de Jager E, Levine AA, Udyavar RN, Burstin HR, Bhulani N, Hoyt DB, Ko CY, Weissman JS, Britt LD, Haider AH, Maggard-Gibbons MA (2019) Disparities in surgical access: a systematic literature review, conceptual model, and evidence map. J Am Coll Surg 228:276. 10.1016/j.jamcollsurg.2018.12.028 30803548 10.1016/j.jamcollsurg.2018.12.028 PMC6391739

[8] Guller U, Jain N, Curtis LH, Oertli D, Heberer M, Pietrobon R (2004) Insurance status and race represent independent predictors of undergoing laparoscopic surgery for appendicitis: secondary data analysis of 145,546 patients. J Am Coll Surg 199:567–575. 10.1016/j.jamcollsurg.2004.06.023. (**discussion 575–577**) 15454140 10.1016/j.jamcollsurg.2004.06.023

[9] Turner M, Adam MA, Sun Z, Kim J, Ezekian B, Yerokun B, Mantyh C, Migaly J (2017) Insurance status, not race, is associated with use of minimally invasive surgical approach for rectal cancer. Ann Surg 265:774–781. 10.1097/SLA.0000000000001781 27163956 10.1097/SLA.0000000000001781

[10] Billmann F, Langan E (2018) Insurance status, not race, is associated with use of minimally invasive surgical approach for rectal cancer. Ann Surg 268:e49. 10.1097/SLA.0000000000002495 28857815 10.1097/SLA.0000000000002495

[11] Aquina CT, Becerra AZ, Justiniano CF, Xu Z, Boscoe FP, Schymura MJ, Noyes K, Monson JRT, Temple LK, Fleming FJ (2019) Surgeon, hospital, and geographic variation in minimally invasive colectomy. Ann Surg 269:1109–1116. 10.1097/SLA.0000000000002694 31082909 10.1097/SLA.0000000000002694

[12] AbuHasan Q, Miller PM, Li WS, Burney CP, Yuce TK, Stefanidis D (2025) Racial disparities in the utilization and outcomes of robotic bariatric surgery: an 8-year analysis of Metabolic and Bariatric Surgery Accreditation Quality Improvement Program data. Surg Obes Relat Dis 21:158–165. 10.1016/j.soard.2024.09.002 39395845 10.1016/j.soard.2024.09.002 PMC11820883

[13] Barnes WA, Carter-Brooks CM, Wu CZ, Acosta DA, Vargas MV (2021) Racial and ethnic disparities in access to minimally invasive gynecologic surgery for benign pathology. Curr Opin Obstet Gynecol 33:279–287. 10.1097/GCO.0000000000000719 34016820 10.1097/GCO.0000000000000719

[14] Hessami K, Leaf M-C, Liang J, Katz A, Chervenak F, AlAshqar A, Borahay MA (2025) Racial disparities in minimally invasive benign hysterectomy. J Soc Laparoendosc Surg 28:e2024.00018. 10.4293/JSLS.2024.00018 10.4293/JSLS.2024.00018 PMC11694781 39749229

[15] Kim SP, Boorjian SA, Shah ND, Weight CJ, Tilburt JC, Han LC, Thompson RH, Trinh Q-D, Sun M, Moriarty JP, Karnes RJ (2013) Disparities in access to hospitals with robotic surgery for patients with prostate cancer undergoing radical prostatectomy. J Urol 189:514–520. 10.1016/j.juro.2012.09.033 23253307 10.1016/j.juro.2012.09.033

[16] Schneider MA, Gero D, Muller M, Horisberger K, Rickenbacher A, Turina M (2021) Inequalities in access to minimally invasive general surgery: a comprehensive nationwide analysis across 20 years. Surg Endosc 35:6227–6343 33206242 10.1007/s00464-020-08123-0 PMC8523463

[17] Erhunmwunsee L, Bhandari P, Sosa E, Sur M, Ituarte PHG, Lui NS (2020) Socioeconomic, rural, and insurance-based inequities in robotic lung cancer resections. Video-Assist Thorac Surg. 10.21037/vats.2020.02.01

[18] Hayanga JWA, Luo X, Hasasna I, Rothenberg P, Reddy S, Mehaffey JH, Lamb J, Badhwar V, Toker A (2024) Intersection of race, rurality, and income in defining access to minimally invasive lung surgery. Ann Thorac Surg. 10.1016/j.athoracsur.2024.03.040 38641193 10.1016/j.athoracsur.2024.03.040 PMC11486839

[19] Fazal ZA, Wall-Wieler E, Patel NM, Johnson S, Yankovsky A, Erhunmwunsee L (2024) Disparities in access to and outcomes of minimally invasive surgery across individual characteristics: a scoping review protocol. OSF. 10.17605/OSF.IO/DH4P3 10.1007/s00464-026-12768-8 PMC13161300 42377518

[20] Tricco AC, Lillie E, Zarin W, O’Brien KK, Colquhoun H, Levac D, Moher D, Peters MDJ, Horsley T, Weeks L, Hempel S, Akl EA, Chang C, McGowan J, Stewart L, Hartling L, Aldcroft A, Wilson MG, Garritty C, Lewin S, Godfrey CM, Macdonald MT, Langlois EV, Soares-Weiser K, Moriarty J, Clifford T, Tunçalp Ö, Straus SE (2018) PRISMA extension for scoping reviews (PRISMA-ScR): checklist and explanation. Ann Intern Med 169:467–473. 10.7326/M18-0850 30178033 10.7326/M18-0850

[21] American College of Surgeons (ACS) (nd) What are the surgical specialties? ACS. https://www.facs.org/for-medical-professionals/education/online-guide-to-choosing-a-surgical-residency/guide-to-choosing-a-surgical-residency-for-medical-students/faqs/specialties/. Accessed 5 Sep 2025

[22] Riner AN, Herremans KM, Deng X, Bandyopadhyay D, Wexner SD, Trevino JG, Sharp SP (2023) Racial/ethnic disparities in the era of minimally invasive surgery for treatment of colorectal cancer. Ann Surg Oncol 30:6748–6759. 10.1245/s10434-023-13693-z 37423924 10.1245/s10434-023-13693-z PMC11235899

[23] Rahimi AO, Soliman D, Hsu CH, Ghaderi I (2024) The impact of gender, race, and ethnicity on bariatric surgery outcomes. Surg Obes Relat Dis 20:454–461. 10.1016/j.soard.2023.12.020 38326184 10.1016/j.soard.2023.12.020

[24] Holland AM, Mead BS, Lorenz WR, Scarola GT, Augenstein VA (2024) Racial and socioeconomic disparities in complex abdominal wall reconstruction referrals. J Abdom Wall Surg 3:12946. 10.3389/jaws.2024.12946 38873344 10.3389/jaws.2024.12946 PMC11169567

[25] Anastasio MK, Spees L, Ackroyd SA, Shih YT, Kim B, Moss HA, Albright BB (2024) Geographic and racial disparities in the quality of surgical care among patients with nonmetastatic uterine cancer. Am J Obstet Gynecol. 10.1016/j.ajog.2024.09.002 39245428 10.1016/j.ajog.2024.09.002

[26] Ramkumar N, Colla CH, Wang Q, O’Malley AJ, Wong SL, Brooks GA (2022) Association of rurality, race and ethnicity, and socioeconomic status with the surgical management of colon cancer and postoperative outcomes among Medicare beneficiaries. JAMA Netw Open 5:e2229247. 10.1001/jamanetworkopen.2022.29247 36040737 10.1001/jamanetworkopen.2022.29247 PMC9428741

[27] Ofshteyn A, Bingmer K, Towe CW, Steinhagen E, Stein SL (2020) Robotic proctectomy for rectal cancer in the US: a skewed population. Surg Endosc 34:2651–2656. 10.1007/s00464-019-07041-0 31372887 10.1007/s00464-019-07041-0

[28] Halloran SJ, Alvarado CE, Sarode A, Sinapoli J, Jiang B, Linden PA, Towe C (2023) Disparities in access to thoracic surgeons among patients receiving lung lobectomy in the United States. Curr Oncol 30:2801–2811. 10.3390/curroncol30030213 36975426 10.3390/curroncol30030213 PMC10047038

[29] Malhotra R, Patel R, Gill K, Brandi KM, Merchant AM (2022) Socioeconomic analysis of the surgical management of ectopic pregnancies: an analysis of the National Inpatient Sample. J Minim Invasive Gynecol 29:641–648. 10.1016/j.jmig.2021.12.020 34995774 10.1016/j.jmig.2021.12.020

[30] Jehan FS, Khreiss M, Seth A, Aziz H (2024) Trends and disparities in access to minimally invasive distal pancreatectomy (MIDP): an eight-year analysis from the National Cancer Database. J Robot Surg 18:52. 10.1007/s11701-023-01775-9 38280048 10.1007/s11701-023-01775-9

[31] Seldomridge AN, Rasic G, Papageorge MV, Ng SC, de Geus SWL, Woods AP, McAneny D, Tseng JF, Sachs TE (2024) Trends in access to minimally invasive pancreaticoduodenectomy for pancreatic cancers. HPB (Oxf) 26:333–343. 10.1016/j.hpb.2023.11.012 10.1016/j.hpb.2023.11.012 38087704

[32] Park JY, Verma A, Tran ZK, Mederos MA, Benharash P, Girgis M (2022) Disparities in utilization and outcomes of minimally invasive techniques for gastric cancer surgery in the United States. Ann Surg Oncol 29:3136–3146. 10.1245/s10434-021-11193-6 34994911 10.1245/s10434-021-11193-6 PMC8990946

[33] Bachelani AM, Holton LA (2024) Factors affecting minimally invasive surgery utilization during elective colectomies for diverticular disease in the United States. Surg Open Sci 19:14–19. 10.1016/j.sopen.2024.03.007 38585039 10.1016/j.sopen.2024.03.007 PMC10995882

[34] Mitzman B, Wang X, Haaland B, Varghese TK (2022) Trends and factors affecting approach choice to pulmonary resection. J Surg Oncol 126:599–608. 10.1002/jso.26923 35603987 10.1002/jso.26923

[35] Chao GF, Montgomery JR, Abou Azar S, Telem DA (2021) Venous thromboembolism: risk factors in the sleeve gastrectomy era. Surg Obes Relat Dis 17:1905–1911. 10.1016/j.soard.2021.06.022 34389247 10.1016/j.soard.2021.06.022

[36] Logan CD, Mahenthiran AK, Siddiqui MR, French DD, Hudnall MT, Patel HD, Murphy AB, Halpern JA, Bentrem DJ (2023) Disparities in access to robotic technology and perioperative outcomes among patients treated with radical prostatectomy. J Surg Oncol 128:375–384. 10.1002/jso.27274 37036165 10.1002/jso.27274 PMC10330024

[37] Tran A, Zheng R, Johnston F, He J, Burns WR, Shubert C, Lafaro K, Burkhart RA (2024) Sociodemographic variation in the utilization of minimally invasive surgical approaches for pancreatic cancer. HPB (Oxf) 26:1280–1290. 10.1016/j.hpb.2024.07.403 10.1016/j.hpb.2024.07.403 PMC11446651 39033045

[38] Traylor J, Simon M, Tsai S, Feinglass J (2020) Patient and hospital characteristics associated with minimally invasive hysterectomy: evidence from 143 Illinois hospitals, 2016 to 2018. J Minim Invasive Gynecol 27:1337–1343. 10.1016/j.jmig.2020.02.013 32126301 10.1016/j.jmig.2020.02.013

[39] Nicola-Ducey L, Nolan O, Cichowski S, Osmundsen B (2024) Racial and ethnic disparities in sacrocolpopexy approach. Urogynecology (Phila) 30:906–918. 10.1097/SPV.0000000000001546 38990736 10.1097/SPV.0000000000001546

[40] Huttler A, Hong C, Shah DK (2022) Racial and ethnic disparities in the surgical management of tubal ectopic pregnancy. F S Rep 3:311–316. 10.1016/j.xfre.2022.08.009 36568938 10.1016/j.xfre.2022.08.009 PMC9783145

[41] Akram WM, Vohra N, Irish W, Zervos EE, Wong J (2022) Racial disparity in the surgical management of diverticular disease. Am Surg 88:929–935. 10.1177/00031348211058623 34964694 10.1177/00031348211058623

[42] Orlando MS, Luna Russo MA, Richards EG, King CR, Park AJ, Bradley LD, Chapman GC (2022) Racial and ethnic disparities in surgical care for endometriosis across the United States. Am J Obstet Gynecol 226:824.e1-824.e11. 10.1016/j.ajog.2022.01.021 35101410 10.1016/j.ajog.2022.01.021

[43] Greenberg AL, Brand NR, Zambeli-Ljepović A, Barnes KE, Chiou SH, Rhoads KF, Adam MA, Sarin A (2023) Exploring the complexity and spectrum of racial/ethnic disparities in colon cancer management. Int J Equity Health 22:68. 10.1186/s12939-023-01883-w 37060065 10.1186/s12939-023-01883-w PMC10105474

[44] Haider SF, Ma S, Xia W, Wood KL, Matabele MM, Quinn PL, Merchant AM, Chokshi RJ (2022) Racial disparities in minimally invasive esophagectomy and gastrectomy for upper GI malignancies. Surg Endosc 36:9355–9363. 10.1007/s00464-022-09210-0 35411463 10.1007/s00464-022-09210-0

[45] Schneyer RJ, Greene NH, Wright KN, Truong MD, Molina AL, Tran K, Siedhoff MT (2022) The impact of race and ethnicity on use of minimally invasive surgery for myomas. J Minim Invasive Gynecol 29:1241–1247. 10.1016/j.jmig.2022.06.025 35793780 10.1016/j.jmig.2022.06.025

[46] Patel R, Pant K, Patel KS, Merchant AM, Alvarez-Downing MM (2022) Association of hospital factors and socioeconomic status with the utilization of minimally invasive surgery for colorectal cancer over a decade. Surg Endosc 36:3750–3762. 10.1007/s00464-021-08690-w 34462866 10.1007/s00464-021-08690-w

[47] Mao J, Genkinger JM, Rundle AG, Wright JD, Schymura MJ, Insaf TZ, Hu JC, Tehranifar P (2024) Robot-assisted surgery and racial and ethnic disparities in post-prostatectomy outcomes among prostate cancer patients. Ann Surg Oncol 31:1373–1383. 10.1245/s10434-023-14447-7 37880515 10.1245/s10434-023-14447-7

[48] Carey ET, Moore KJ, McClurg AB, Degaia A, Tyan P, Schiff L, Dieter AA (2023) Racial disparities in hysterectomy route for benign disease: examining trends and perioperative complications from 2007 to 2018 using the NSQIP database. J Minim Invasive Gynecol 30:627–634. 10.1016/j.jmig.2023.03.024 37037283 10.1016/j.jmig.2023.03.024

[49] Kim JS, Qureshy Z, Lazar AA, Chen LL, Jacoby A, Opoku-Anane J, Lager J (2022) Rethinking disparities in minimally invasive myomectomy: identifying drivers of disparate surgical approach to myomectomy between African American and White women. J Minim Invasive Gynecol 29:65-71.e2. 10.1016/j.jmig.2021.06.016 34192565 10.1016/j.jmig.2021.06.016

[50] Boyd Brittni AJ, Winkelman WD, Mishra K, Vittinghoff E, Jacoby VL (2021) Racial and ethnic differences in reconstructive surgery for apical vaginal prolapse—PubMed. Am J Obstet Gynecol. 10.1016/j.ajog.2021.05.002 33984303 10.1016/j.ajog.2021.05.002

[51] Wood KL, Haider SF, Bui A, Leitman IM (2020) Access to common laparoscopic general surgical procedures: do racial disparities exist? Surg Endosc 34:1376–1386. 10.1007/s00464-019-06912-w 31209603 10.1007/s00464-019-06912-w

[52] Su W-TK, Coleman CM, Bossick AS, Lee-Griffith M, Wegienka G (2022) Racial differences in planned hysterectomy procedure route. J Womens Health (Larchmt) 31:31–37. 10.1089/jwh.2021.0132 34637634 10.1089/jwh.2021.0132

[53] Pollack LM, Olsen MA, Gehlert SJ, Chang S-H, Lowder JL (2020) Racial/ethnic disparities/differences in hysterectomy route in women likely eligible for minimally invasive surgery. J Minim Invasive Gynecol 27:1167-1177.e2. 10.1016/j.jmig.2019.09.003 31518712 10.1016/j.jmig.2019.09.003 PMC7062558

[54] Porras Fimbres DC, Nussbaum DP, Mosca PJ (2023) Racial disparities in time to laparoscopic cholecystectomy for acute cholecystitis. Am J Surg 226:261–270. 10.1016/j.amjsurg.2023.05.004 37149406 10.1016/j.amjsurg.2023.05.004

[55] Barbaresso R, Qasba N, Knee A, Benabou K (2024) Racial disparities in surgical treatment of uterine fibroids during the COVID-19 pandemic. J Womens Health (Larchmt) 33:1085–1094. 10.1089/jwh.2023.0826 38629437 10.1089/jwh.2023.0826

[56] Haider S, Wood K, Bui A, Leitman IM (2021) Racial disparities in outcomes after common abdominal surgical procedures—the impact of access to a minimally invasive approach. J Surg Res 257:85–91. 10.1016/j.jss.2020.07.056 32818788 10.1016/j.jss.2020.07.056

[57] DeAngelis EJ, Zebley JA, Ileka IS, Ganguli S, Panahi A, Amdur RL, Vaziri K, Lee J, Jackson HT (2023) Trends in utilization of laparoscopic colectomy according to race: an analysis of the NIS database. Surg Endosc 37:1421–1428. 10.1007/s00464-022-09381-w 35731300 10.1007/s00464-022-09381-w

[58] Summey RM, Pike J, Salazar C (2022) Postoperative risks for Hispanic patients undergoing hysterectomy for benign indications. J Racial Ethn Health Disparities 9:684–690. 10.1007/s40615-021-01001-y 33646554 10.1007/s40615-021-01001-y

[59] Zaritsky E, Le A, Tucker LY, Ojo A, Weintraub MR, Raine-Bennett T (2022) Minimally invasive myomectomy: practice trends and differences between Black and non-Black women within a large integrated healthcare system. Am J Obstet Gynecol 226:826 e1-826 e11. 10.1016/j.ajog.2022.01.022 35101407 10.1016/j.ajog.2022.01.022

[60] Johnson M, Carreño PK, Lutgendorf MA, Brown JE, Velosky AG, Highland KB (2023) Hysterectomy inequities between black and white patients in the US military health system: a retrospective cohort study. Eur J Obstet Gynecol Reprod Biol 286:52–60. 10.1016/j.ejogrb.2023.05.006 37209523 10.1016/j.ejogrb.2023.05.006

[61] Rios EM, Parma MA, Fernandez RA, Clinton TN, Reyes RM, Kaushik D, Pruthi D, Mansour AM, Mukherjee N, Gelfond J, Wheeler KM, Svatek RS (2020) Urinary diversion disparity following radical cystectomy for bladder cancer in the Hispanic population. Urology 137:66–71. 10.1016/j.urology.2019.12.017 31883879 10.1016/j.urology.2019.12.017 PMC7063861

[62] Amirian H, Torquati A, Omotosho P (2020) Racial disparity in 30-day outcomes of metabolic and bariatric surgery. Obes Surg 30:1011–1020. 10.1007/s11695-019-04282-9 31745861 10.1007/s11695-019-04282-9 PMC7222128

[63] Edwards MA, Coombs S, Spaulding A (2022) Racial disparity in causes for readmission following bariatric surgery. Surg Obes Relat Dis 18:241–252. 10.1016/j.soard.2021.10.015 34863671 10.1016/j.soard.2021.10.015

[64] Edwards MA, Sarvepalli S, Mazzei M, Acevedo E Jr, Lu X, Zhao H (2020) Outcomes in racial and ethnic minorities after revisional robotic-assisted metabolic and bariatric surgery: an analysis of the MBSAQIP database. Surg Obes Relat Dis 16:1929–1937. 10.1016/j.soard.2020.08.019 33036945 10.1016/j.soard.2020.08.019

[65] Agarwal S, Bruff A, Mazzei M, Zhao H, Edwards MA (2021) Exploring racial disparity in perioperative outcomes following revisional bariatric surgery: a case–control matched analysis. Am J Surg 221:741–748. 10.1016/j.amjsurg.2020.03.030 32279831 10.1016/j.amjsurg.2020.03.030

[66] Falagario UG, Martini A, Pfail J, Treacy PJ, Okhawere KE, Dayal BD, Sfakianos JP, Abaza R, Eun DD, Bhandari A, Porter JR, Hemal AK, Badani KK (2020) Does race impact functional outcomes in patients undergoing robotic partial nephrectomy? Transl Androl Urol 9:863–869. 10.21037/tau.2019.09.31 32420201 10.21037/tau.2019.09.31 PMC7214979

[67] Acevedo E, Lu X, Zhao H, Mazzei M, Sarvepalli S, Edwards MA (2021) Outcomes in racial minorities after robotic Roux-en-Y gastric bypass and sleeve gastrectomy: a retrospective review of the Metabolic and Bariatric Surgery Accreditation and Quality Improvement Program database. Surg Obes Relat Dis. 10.1016/j.soard.2020.10.019 33257274 10.1016/j.soard.2020.10.019

[68] Kahveci AC, Dooley MJ, Johnson J, Mund AR (2024) Are there racial disparities in perioperative pain? A retrospective study of a gynecological surgery cohort. J Perianesth Nurs 39:82–86. 10.1016/j.jopan.2023.06.097 37855762 10.1016/j.jopan.2023.06.097 PMC10873002

[69] Berman JM, Bradley L, Hawkins SM, Levy B (2022) Uterine fibroids in Black women: a race-stratified subgroup analysis of treatment outcomes after laparoscopic radiofrequency ablation. J Womens Health (Larchmt) 31:593–599. 10.1089/jwh.2020.9001 34287028 10.1089/jwh.2020.9001 PMC9063135

[70] Alanee S, Chammout D, Deebajah M, Peabody J, Menon M (2022) Association of request for opioid medications refill after hospital discharge with race in patients with prostate cancer treated with robotic-assisted laparoscopic radical prostatectomy. J Opioid Manag 18:447–453. 10.5055/jom.2022.0738 36226784 10.5055/jom.2022.0738

[71] Meyer R, Siedhoff M, Truong M, Hamilton K, Fan S, Levin G, Barnajian M, Nasseri Y, Wright K (2023) Risk factors for major complications following minimally invasive surgeries for endometriosis in the United States. J Minim Invasive Gynecol 30:820–826. 10.1016/j.jmig.2023.06.002 37321298 10.1016/j.jmig.2023.06.002

[72] Sands KG, Bhatt R, Vetter J, Paradis A, Chow AK, Bhayani S, Figenshau RS, Venkatesh R (2021) Racial comparison of patients undergoing minimally invasive partial nephrectomy for renal masses at a large volume tertiary center. J Endourol 35:1365–1371. 10.1089/end.2020.0655 33730861 10.1089/end.2020.0655

[73] Sundaresan N, Roberts A, Thompson KJ, McKillop IH, Barbat S, Nimeri A (2020) Examining the Hispanic paradox in bariatric surgery. Surg Obes Relat Dis 16:1392–1400. 10.1016/j.soard.2020.06.009 32694042 10.1016/j.soard.2020.06.009

[74] Meyer R, Nasseri YY, Barnajian M, Siedhoff MT, Wright KN, Hamilton KM, Levin G, Truong MD (2024) Risk factors for major complications following colorectal resections for endometriosis in the USA. Int J Colorectal Dis. 10.1007/s00384-023-04577-5 10.1007/s00384-023-04577-5 PMC10700479 38055072

[75] Ajay D, Li H, Barrett-Harlow B, Nguyen J, Benson C, Wang X, Chapin BF, Davis J, Westney OL (2022) Perioperative factors contributing to delayed return of continence after radical prostatectomy: the role of race and comorbidities. Continence. 10.1016/j.cont.2022.100496

[76] Barrington DA, Meade CE, Cosgrove CM, Cohn DE, Felix AS (2022) Racial and ethnic disparities in readmission risk following the surgical management of endometrial cancer. Gynecol Oncol 166:543–551. 10.1016/j.ygyno.2022.07.014 35882610 10.1016/j.ygyno.2022.07.014

[77] Ko JS, Suh CH, Huang H, Zhuo H, Harmanli O, Zhang Y (2021) Association of race/ethnicity with surgical route and perioperative outcomes of hysterectomy for leiomyomas. J Minim Invasive Gynecol 28:1403-1410.e2. 10.1016/j.jmig.2020.11.008 33242598 10.1016/j.jmig.2020.11.008

[78] Edwards MA, Hussain MWA, Spaulding AC (2023) Gastric bypass mortality trends in racial cohorts: are we improving? Obes Surg 33:1411–1421. 10.1007/s11695-023-06541-2 36918474 10.1007/s11695-023-06541-2

[79] Matabele MM, Haider SF, Wood Matabele KL, Merchant AM, Chokshi RJ (2023) The mediating effect of operative approach on racial disparities in bariatric surgery complications. J Surg Res 289:42–51. 10.1016/j.jss.2023.03.026 37084675 10.1016/j.jss.2023.03.026

[80] Rios EM, Parma MA, Fernandez RA, Clinton TN, Kaushik D, Pruthi D (2019) Urinary diversion disparity following radical cystectomy for bladder cancer in the Hispanic population. Urology 137:66–71 31883879 10.1016/j.urology.2019.12.017 PMC7063861

[81] Stodolski M, Zirngibl H, Ambe PC (2020) Gender discrimination in endoscopic groin hernia repair. Minimal invasive groin hernia repair is offered less often to female patients compared to male patients. J Visc Surg 157:271–276. 10.1016/j.jviscsurg.2019.12.006 31870628 10.1016/j.jviscsurg.2019.12.006

[82] Howard R, Ehlers A, Delaney L, Solano Q, Shen M, Englesbe M, Dimick J, Telem D (2023) Sex disparities in the treatment and outcomes of ventral and incisional hernia repair. Surg Endosc 37:3061–3068. 10.1007/s00464-022-09475-5 35920905 10.1007/s00464-022-09475-5

[83] Dugan N, Thompson KJ, Barbat S, Prasad T, McKillop IH, Maloney SR, Roberts A, Gersin KS, Kuwada TS, Nimeri A (2020) Male gender is an independent risk factor for patients undergoing laparoscopic sleeve gastrectomy or Roux-en-Y gastric bypass: an MBSAQIP® database analysis. Surg Endosc 34:3574–3583. 10.1007/s00464-019-07106-0 32072290 10.1007/s00464-019-07106-0 PMC7224103

[84] Howell EC, Sakai-Bizmark R, Karunungan K, Pak Y, Ugarte R, Richardson S, DeUgarte DA, Lee SL (2024) Disparities in outpatient rural cholecystectomy outcomes. Am J Surg 236:115852. 10.1016/j.amjsurg.2024.115852 39106552 10.1016/j.amjsurg.2024.115852 PMC11681941

[85] Childers CP, Uppal A, Tillman M, Chang GJ, Tran Cao HS (2023) Insurance disparities in access to robotic surgery for colorectal cancer. Ann Surg Oncol 30:3560–3568. 10.1245/s10434-023-13354-1 36943527 10.1245/s10434-023-13354-1

[86] Lee Y, Andrew L, Hill S, An KR, Chatroux L, Anvari S, Hong D, Kuhnen AH (2023) Disparities in access to minimally invasive surgery for inflammatory bowel disease and outcomes by insurance status: analysis of the 2015 to 2019 National Inpatient Sample. Surg Endosc 37:9420–9426. 10.1007/s00464-023-10400-7 37679584 10.1007/s00464-023-10400-7

[87] Branche C, Sakowitz S, Porter G, Cho NY, Chervu N, Mallick S, Bakhtiyar SS, Benharash P (2024) Utilization of minimally invasive colectomy at safety-net hospitals in the United States. Surgery 176:172–179. 10.1016/j.surg.2024.03.036 38729887 10.1016/j.surg.2024.03.036

[88] Hrebinko KA, Rieser C, Nassour I, Tohme S, Sabik LM, Khan S, Medich DS, Zureikat AH, Hoehn RS (2021) Patient factors limit colon cancer survival at safety-net hospitals: a national analysis. J Surg Res 264:279–286. 10.1016/j.jss.2021.03.012 33839343 10.1016/j.jss.2021.03.012 PMC8614240

[89] Eguia E, Baker MS, Chand B, Sweigert PJ, Kuo PC (2020) The impact of the Affordable Care Act (ACA) Medicaid expansion on access to minimally invasive surgical care. Am J Surg 219:15–20. 10.1016/j.amjsurg.2019.07.003 31307661 10.1016/j.amjsurg.2019.07.003 PMC6917909

[90] Dingillo G, Alvarado CE, Rice JD, Sinopoli J, Badrinathan A, Linden PA, Towe CW (2023) Affordable Care Act Medicaid expansion is associated with increased utilization of minimally invasive lung resection for early stage lung cancer. Am Surg 89:5147–5155. 10.1177/00031348221138081 36341749 10.1177/00031348221138081

[91] Badrinathan A, Syphan TA, Bassiri A, Linden J, Alvarado CE, Sinopoli J, Tapias Vargas L, Towe CW (2023) The impact of disparities on minimally invasive esophagectomy after the 2014 Affordable Care Act expansion: a retrospective analysis. Foregut 3:199–207. 10.1177/26345161231178351

[92] Liu N, Venkatesh M, Hanlon BM, Muraveva A, Johnson MK, Hanrahan LP, Funk LM (2021) Association between Medicaid status, social determinants of health, and bariatric surgery outcomes. Ann Surg Open 2:e028. 10.1097/as9.0000000000000028 33912867 10.1097/AS9.0000000000000028 PMC8059876

[93] Wu J, Silva JP, Toriola T, Palmer RC, Hernandez F, Compton E, Abel S, Nguyen JD, Dobrowolsky A, Martin MJ, Samakar K (2022) Evaluating the bariatric safety net: analysis of socioeconomic factors and outcomes at a bariatric safety net program compared to an affiliated private center. Obes Surg 32:3973–3983. 10.1007/s11695-022-06307-2 36198928 10.1007/s11695-022-06307-2

[94] Dugan MM, Ross SB, Sucandy I, Slavin M, Pattilachan TM, Christodoulou M, Rosemurgy A (2024) Cost comparison between Medicare and private insurance for robotic transhiatal esophagectomy. J Robot Surg 18:30. 10.1007/s11701-023-01762-0 38231356 10.1007/s11701-023-01762-0

[95] Jermihov A, Chen L, Echavarria MF, Ng EP, Velez FO, Moodie CC, Garrett JR, Fontaine JP, Toloza EM (2022) Effect of socio-economic status on perioperative outcomes after robotic-assisted pulmonary lobectomy. Cureus 14:e26201. 10.7759/cureus.26201 35754434 10.7759/cureus.26201 PMC9224841

[96] Johns AJ, Luce MS, Kaneski MJ, Lowery RA, Jachniewicz B, Salas A, McCreary R, Russell RM, Lyo V, Ali MR, Ahmed SM (2023) The long weight: association between distressed communities index and long-term weight outcomes following bariatric surgery. Surg Endosc 37:7218–7225. 10.1007/s00464-023-10158-y 37369948 10.1007/s00464-023-10158-y PMC10462541

[97] Tracy BM, Finnegan TM, Smith RN, Senkowski CK (2021) Random forest modeling using socioeconomic distress predicts hernia repair approach. Surg Endosc 35:3890–3895. 10.1007/s00464-020-07860-6 32757067 10.1007/s00464-020-07860-6

[98] Halloran SJ, Alvarado CE, Sarode AL, Jiang B, Sinopoli J, Linden PA, Towe CW (2023) Disparities in access to thoracic surgeons among patients receiving lung lobectomy in the United States. Curr Oncol 30:2801–2811. 10.3390/curroncol30030213 36975426 10.3390/curroncol30030213 PMC10047038

[99] Sakowitz S, Bakhtiyar SS, Curry J, Ali K, Toste P, Benharash P (2024) Association of neighborhood socioeconomic disadvantage with use of minimally invasive resection for non-small cell lung cancer. J Thorac Cardiovasc Surg 168:1270-1280.e1. 10.1016/j.jtcvs.2023.12.008 38101767 10.1016/j.jtcvs.2023.12.008

[100] Bakillah E, Sharpe J, Tong JK, Goldshore M, Morris JB, Kelz RR (2023) Non-English primary language: a growing population’s access to cholecystectomy. Ann Surg 278:e1175–e1179. 10.1097/sla.0000000000005919 37226825 10.1097/SLA.0000000000005919

[101] Kosber RL, Ha AS, Kurtzman JT, Blum R, Brandes SB (2022) Hospital ownership, geographic region, patient age, comorbidities, and insurance status appear to influence patient selection robot-assisted ureteral reimplantation for benign disease: a population-based analysis. J Endourol 36:224–230. 10.1089/end.2021.0415 34278805 10.1089/end.2021.0415

[102] González Peña T, Jesse NJ, Zhao Z, Harvey LFB, Fajardo OM (2025) Language-based disparities in route of hysterectomy for benign disease. J Minim Invasive Gynecol 32:151–158. 10.1016/j.jmig.2024.09.013 39305983 10.1016/j.jmig.2024.09.013

[103] Joo H, Fernández A, Wick EC, Moreno Lepe G, Manuel SP (2023) Association of language barriers with perioperative and surgical outcomes: a systematic review. JAMA Netw Open 6:e2322743. 10.1001/jamanetworkopen.2023.22743 37432686 10.1001/jamanetworkopen.2023.22743 PMC10336626

[104] Martinez RAM, Andrabi N, Goodwin AN, Wilbur RE, Smith NR, Zivich PN (2023) Conceptualization, operationalization, and utilization of race and ethnicity in major epidemiology journals, 1995–2018: a systematic review. Am J Epidemiol 192:483–496. 10.1093/aje/kwac146 35938872 10.1093/aje/kwac146 PMC9985111

[105] Darzi SA, Munz Y (2004) The impact of minimally invasive surgical techniques. Annu Rev Med 55:223–237. 10.1146/annurev.med.55.091902.105248 14746519 10.1146/annurev.med.55.091902.105248

[106] Kawka M, Fong Y, Gall TMH (2023) Laparoscopic versus robotic abdominal and pelvic surgery: a systematic review of randomised controlled trials. Surg Endosc 37:6672–6681. 10.1007/s00464-023-10275-8 37442833 10.1007/s00464-023-10275-8 PMC10462573

