# Supplementary material for: Disparities in access to and outcomes of minimally invasive surgery: a scoping review
Source: Surg Endosc. 2026 Apr 2;40(5):3628–40. doi: 10.1007/s00464-026-12768-8 (PMC13161300; doi:10.1007/s00464-026-12768-8)
Supplement: Supplementary file 2 — Supplementary file2 (DOCX 1699 kb) [file 464_2026_12768_MOESM2_ESM.docx]

**APPENDIX**Supplemental Table 2: Studies included within race/ethnicity domain

| **Type of outcome reported** | **Method of disparity analysis** | **Author, year** | **Study design** | **Surgical specialty and procedure** | **Form of MIS included** | **Sample size of patients** | **Findings** | **doi** |
| --- | --- | --- | --- | --- | --- | --- | --- | --- |
| Papers that reported disparities in outcomes | Single dimension of disparity analyzed | Amirian, 2020 | Retrospective Cohort | General surgery (Bariatrics - RYGB and SG) | Lap only | 106932 | African American race was associated with a higher risk of postoperative complications (odds ratio [OR] 1.13; confidence interval [CI] 1.06–1.2) and readmissions (OR 1.47; CI 1.3–1.6). While American Indian or Alaska Native were also associated with higher re-interventions (OR 2.11; CI1.03–4.3) | 10.1007/s11695-019-04282-9 |
|  |  | Edwards, 2022 | Retrospective Cohort | General surgery (Bariatrics - RYGB and SG) | Lap only | 550671 | Black race was independently associated with readmission (P <.05). | 10.1016/j.soard.2021.10.015 |
|  |  | Mao, 2024 | Retrospective Cohort | Urology (Radical prostatectomy) | RAS only | 18926 | NHB and Hispanic patients had lower utilization of RARP and higher risks of postoperative adverse events than NHW patients. | 10.1245/s10434-023-14447-7 |
|  |  | Edwards, 2020 | Retrospective Cohort | General surgery (Bariatrics) | RAS only | 1922 | After propensity matching, outcomes between black and white patients were similar, except for higher rates of superficial surgical site infection among white patients (P =.05) and higher rates of organ space surgical site infection in black patients (P =.05). | 10.1016/j.soard.2020.08.019 |
|  |  | Agarwal, 2021 | Retrospective Cohort | General surgery (Revisional bariatrics) | Lap & RAS | 27030 | Operative duration (p = 0.008) and length of stay (p ¼ 0.0003) were longer in Black patients. Readmission (6.8% vs. 5.4%, p = 0.009) was higher in Black patients. Bleeding (0.82% vs.0.38%, p = 0.02) and surgical site infection (SSI) (2.6% vs. 1.8%, p = 0.01) were higher in White patients. | 10.1016/j.amjsurg.2020.03.030 |
|  |  | Falagario, 2020 | Retrospective Cohort | Urology (partial nephrectomy) | RAS only | 999 | In multivariate analysis, AA race did not emerge as a significant factor for predicting acute kidney injury scores (OR 1.10, P=0.8). | 10.21037/tau.2019.09.31 |
|  |  | Acevedo, 2021 | Case-control | General surgery (Bariatrics - RYGB) | RAS only | 2666 | Black RYGB patients had longeroperative lengths (OLs; P 5.0008) and postoperative lengths of stay (P 5.001), and a higher rate ofpulmonary embolism (P 5 .05). Hispanic (versus White) RYGB patients had longer lengths of stay(P 5 .007). | 10.1016/j.soard.2020.10.019 |
|  |  | Kahveci 2024 | Retrospective Cohort | Gynecology (Hysterectomy) | Lap, RAS, vaginal | 203 | In this specific population, there was no evidence of racial disparities in postoperative pain or intraoperative analgesia administration. | 10.1016/j.jopan.2023.06.097 |
|  |  | Berman 2022 | Retrospective Cohort | Gynecology (Myomectomy) | Lap only | 135 | Menstrual blood loss (140.1 mL vs.127.02 mL; P = 0.44) and mean fibroid volume reduction (47.5 cm3 vs. 36.0 cm 3; P = 0.17) were similar betweenBlack and White women at 12 months. | 10.1089/jwh.2020.9001 |
|  |  | Alanee 2022 | Retrospective Cohort | Urology (prostatectomy) | RAS and lap | 282 | No racial variations in request of refills were identified for patients undergoing MIS | 10.5055/jom.2022.0738 |
|  |  | Meyer 2023 | Retrospective Cohort | Gynecology (endometriosis) | RAS and lap | 28697 | African American race is a risk factor for majorcomplications among women undergoing surgeries with and without bowel procedures or hysterectomy | 10.1016/j.jmig.2023.06.002 |
|  |  | Sands 2021 | Retrospective Cohort | Urology (partial nephrectomy) | RAS and lap | 981 | There were no differences in 30-day complications ( p = 0.330) and by using Kaplan–Meier curves,there was no observed difference in overall survival ( p = 0.752) or disease-free survival ( p = 0.403) for Black and White patients undergoing MIS | 10.1089/end.2020.0655 |
|  |  | Sundaresan 2020 | Retrospective Cohort | General surgery (Bariatrics - RYGB and SG) | Lap only | 388652 | Rates of major and minor complications in Hispanic patients were lower in the Roux-en-Y gastric bypass group. Similarly, there were no differences between Hispanic/non-Hispanic sleeve gastrectomy patients in overall major/minor complication rates | 10.1016/j.soard.2020.06.009 |
|  |  | Meyer 2024 | Retrospective Cohort | General Surgery (colon resection) | Lap only | 755 | Hispanic ethnicity was associated with occurrence of major complications after colorectal resection in patients with endometriosis | 10.1007/s00384-023-04577-5 |
|  |  | Ajay 2022 | Retrospective Cohort | Urology (radical prostatectomy) | RAS only | 1275 | Black race (HR 0.77, 𝑝 0.04) was found to be a predictor for delay in drying and worse outcome for urinary continence after RAS | 10.1016/j.cont.2022.100496 |
|  | Intersectional dimensions of disparity analyzed | Rahimi, 2024 | Retrospective cohort | General surgery (bariatrics - SG, RYGB and duodenal switch) | Lap only | 975642 | Both females and males NHBs had higher odds of postoperative com-plications compared to NHWs in sleeve gastrectomy (Female aOR:1.31, 95% CI: [1.23–1.40]; MaleaOR:1.24, 95% CI: [1.08–1.43], P , .001) and gastric bypass (Female aOR:1.24, 95% CI: [1.16–1.33]; Male aOR:1.25, 95% CI: [1.06–1.48], P , .01) | 10.1016/j.soard.2023.12.020 |
|  | Several dimensions of disparity analyzed | Chao, 2021 | Retrospective cohort | General surgery (bariatrics - SG and RYGB) | Lap only | 605782 | On a multivariable analysis, black race was found to be significant risk factor for postoperative VTE | 10.1016/j.soard.2021.06.022 |
| Papers that reported disparities in access | Single dimension of disparity analyzed | Nicola-Ducey, 2024 | Cross sectional | Urogynecology (Sacrocolpopexy) | Lap & RAS | 41,837 | Black (aOR 2, 95% CI 1.26–3.16,P < 0.003) and Hispanic patients (aOR 1.73,95% CI 1.31–2.28, P < 0.001) were more likely to undergo abdominal sacrocolpopexy compared to White patients | 10.1097/spv.0000000000001546 |
|  |  | Huttler, 2022 | Retrospective Cohort | Gynecology (Salpingectomy and Salpingostomy) | Lap only | 7,791 | Odds of undergoing laparoscopic surgery were lower in Black (adjusted odds ratio [aOR] 0.52; 95%CI, 0.45–0.61) and Hispanic patients (aOR 0.52; 95% CI, 0.44–0.61) compared with White patients | 10.1016/j.xfre.2022.08.009 |
|  |  | Akram, 2022 | Retrospective Cohort | General Surgery (colectomy) | Lap & RAS | 46,713 | After adjusting for confounders, black race was associated with open surgery P < .0001 | 10.1177/00031348211058623 |
|  |  | Orlando, 2022 | Retrospective Cohort | Gynecology (Hysterectomy and non-hysterectomy) | Lap only | 11,936 | Compared with White patients, all racial and ethnic groups other than American Indian or Alaska Natives were more likely to undergo abdominal rather than minimally invasive hysterectomy (Hispanic: OR, 1.70[CI, 1.39e2.06]; Black or AfricanAmerican: OR, 1.93 [CI, 1.57e2.36];Asian: OR, 2.03 [CI, 1.56e2.65]; NativeHawaiian or Pacific Islander: OR, 5.16[CI, 2.67e9.95]; and unknown race or ethnicity: OR, 2.15 [CI, 1.82e2.55]). | 10.1016/j.ajog.2022.01.021 |
|  |  | Greenberg, 2023 | Retrospective Cohort | General Surgery (transverse, left, and rectosigmoid colon) | Lap & RAS | 326,003 | Hispanic/Spanish (OR 1.08,p < 0.01), East Asian (OR 1.21, p < 0.01), and South Asian(OR 1.21, p = 0.01) patients were more likely to have surgery with an MIS technique than White patients. No significant differences in likelihood of MIS for Black, American Indian, Aleutian, and Eskimo, Native Hawaiian and Other Pacific Island and Southeast Asian patients when compared to white patients | 10.1186/s12939-023-01883-w |
|  |  | Haider, 2022 | Retrospective Cohort | Cardiothoracic (Esophagectomy and Gastrectomy) | Lap only | 13,023 | Black self-reported race is an independent predictor of open approach to gastrectomy (OR1.6871943, 95% CI 1.431464–1.989829, p < 0.001). Black self-reported race was not predictive of operative approach among esophagectomy patients (OR 0.7942576, 95% CI 0.5698645–1.124228, p = 0.183) | 10.1007/s00464-022-09210-0 |
|  |  | Boyd, 2021 | Retrospective Cohort | Urogynecology (Sacrocolpopexy) | Lap only | 22,861 | Latina women and Native Hawaiian or Pacific Islander women were less likely to undergo a laparoscopic approach sacrocolpopexy compared with White women (OR, 0.68; 95% CI 0.58-0.79, and OR, 0.31; 95% CI, 0.1-0.56) | 10.1016/j.ajog.2021.05.002 |
|  |  | Wood, 2020 | Retrospective Cohort | General Surgery (inguinal hernia repair, cholecystectomy, appendectomy, and colectomy) | Lap only | 213501.00 | Overall, patients of non-white race/ethnicity had a lower incidence of minimally-invasive approach to surgery. On univariate analysis, black racewas significantly associated with undergoing an open approach in all four surgical procedures under investigation | 10.1007/s00464-019-06912-w |
|  |  | Su, 2022 | Prospective Cohort | Gynecology (Hysterectomy) | Lap & Vaginal | 431.00 | After adjusting for confounders, uterine weight and a surgical indication of fibroids, there was no difference in the likelihood of having planned an MIS hysterectomy between Black women and White women | 10.1089/jwh.2021.0132 |
|  |  | Pollack, 2020 | Cross sectional | Gynecology (Hysterectomy) | Lap & Vaginal | 133,082 | African American and Hispanic women were less likely to undergo vaginal (aPR 0.93,95% CI 0.90–0.96 and aPR 0.95, 95% CI 0.93–0.97, respectively) and laparoscopic hysterectomy(aPR 0.90, 95% CI 0.87–0.94 and aPR 0.95, 95% CI 0.92–0.98, respectively) than White women | 10.1016/j.jmig.2019.09.003 |
|  |  | Porras Fimbres, 2023 | Retrospective Cohort | General Surgery (Cholecystectomy) | Lap only | 121,466 | In the multivariable analysis, controlling for potential confounding factors, Black patients were found to be more likely than White patients to experience a time to surgery >1 day (OR1.23, 95% CI 1.17–1.30, p < 0.0001) | 10.1016/j.amjsurg.2023.05.004 |
|  |  | Barbaresso,, 2024 | Cross sectional | Gynecology (Hysterectomy) | Lap, Vaginal, & RAS | 350.00 | Minimally invasive hysterectomy occurred more frequently among White patients than non-White patients (7.5% points higher [95% confidenceinterval (CI) = -3.1 to 18.2]), | 10.1089/jwh.2023.0826 |
|  |  | Haider, 2021 | Retrospective Cohort | Cardiothoracic (Esophagectomy and gastrectomy) | Lap only | 7,891 | Black self-reported race is an independent predictor of open approach to gastrectomy (OR1.6871943, 95% CI 1.431464–1.989829, p < 0.001). | 10.1016/j.jss.2020.07.056 |
|  |  | Schneyer, 2022 | Retrospective Cohort | Gynecology (Hysterectomy and Myomectomy) | Lap, Vaginal, & RAS | 1,311 | Black and Hispanic patients were less likely to undergo MIS vs white patients (aOR 0.33, 95% CI 0.22−0.48 and aOR 0.44, 95% CI 0.28−0.72, respectively) | 10.1016/j.jmig.2022.06.025 |
|  |  | Mao, 2024 | Retrospective Cohort | Urology (prostatectomy) | RAS only | 18,926 | Hispanic and non-Hispanic Black patients were less likely to receive RARP than non-Hispanic White patients (OR = 0.78, 95% CI = 0.62 to 0.98; OR = 0.75, 95% CI = 0.57 to 1.00, respectively) | 10.1093/jncics/pkae061 |
|  |  | Carey, 2023 | Retrospective Cohort | Gynecology (Hysterectomy) | Lap, Vaginal, & RAS | 269,791 | For each year from 2007 to 2018, the proportion of women undergoing open abdominal hysterectomy remained twice as high in Black Women compared with White women (33.1%−14.4%, p <.01). | 10.1016/j.jmig.2023.03.024 |
|  |  | Kim, 2022 | Retrospective Cohort | Gynecology (myomectomy) | Lap & RAS | 386.00 | African American women went through MIS myomectomy at similar rates as White women when adjusting for all relevant confounding variables (aOR 1.3; 95% CI, 0.8−2.2 myomas;p <.01). | 10.1016/j.jmig.2021.06.016 |
|  |  | DeAngelis, 2023 | Retrospective Cohort | General Surgery (colectomy) | Lap only | 267,856 | After adjusting for covariates, Black patients had significantly lower adjusted odds of undergoing laparoscopic colectomy vs White patients (aOR 0.92, p < 0.0001). | 10.1007/s00464-022-09381-w |
|  |  | Summey, 2022 | Retrospective Cohort | Gynecology (Hysterctomy) | Lap &vaginal | 102051.00 | Hispanic patients were more likely to undergo abdominal hysterectomy(30.0 vs 19.1%, p < 0.01) | 10.1007/s40615-021-01001-y |
|  |  | Zaritsky, 2022 | Retrospective Cohort | Gynecology (myomectomy) | Lap and RAS | 4033.00 | The proportion of minimally invasive myomectomy in Black women remained lower than in non-Black women (54.5% vs 64.7%; P<.001) | 10.1016/j.ajog.2022.01.022 |
|  |  | Matushita, 2020 | Retrospective Cohort | Gynecology (myomectomy) | Lap only | 11633.00 | African American population showed a significantly greater increase in the odds of abdominal myomectomy over laparoscopic myomectomy in comparison with the white population (1.22; 95% CI, 1.02−1.47; p = .03). | 10.1016/j.jmig.2019.03.019 |
|  |  | Johnson, 2023 | Retrospective Cohort | Gynecology (hysterectomy) | Lap & vaginal | 11067.00 | Black patients were more likely to receive an open hysterectomy [log(OR) 0.54, (95 %CI 0.65, 0.43), p < 0.001]. | 10.1016/j.ejogrb.2023.05.006 |
|  |  | Rios, 2020 | Retrospective Cohort | Urology (radical cystectomy) | Lap only | 507.00 | There was no significant association of surgical approach (i.e., laparoscopic versus open) across ethnicity (Hispanic vs non-Hispanic patients). | 10.1016/j.urology.2019.12.017 |
|  | Intersectional dimensions of disparity analyzed | Hayanga, 2024 | Retrospective Cohort | Cardiothoracic (lobectomy) | RAS only | 13,403 | Low-income Black/urban patients had higher RATS (P [ .002), and fewer open resections (P < .001) compared with rural White patients (P [ .005) | 10.1016/j.athoracsur.2024.03.040 |
|  |  | Riner, 2023 | Cross sectional | General Surgery (Colorectal resection) | Lap and RAS | 216,364 | Surgical approach significantly differed by race and ethnicity (p < 0.0001), with the highest rate of open surgery among Non Hispanic Black patients | 10.1245/s10434-023-13693-z |
|  | Several dimensions of disparity analyzed | Patel, 2022 | Retrospective Cohort | General surgery (colectomy) | Lap & RAS | 82,965 | Black patients were associated with decreased odds of MIS use during hospitalization compared to White patients (OR 0.921, p = 0.0011). | 10.1007/s00464-021-08690-w |
|  |  | Anastasio, 2024 | Cross sectional | Gynecology (Hysterectomy) | Lap & RAS | 21,837 | When compared with non-Black patients, Black patients were less likely to undergo minimally invasive surgery (57.0% vs 74.1%). In adjusted regression models that controlled for a diagnosis of fibroids, Black race was an independent risk factor for the receipt of open surgery. | 10.1016/j.ajog.2024.09.002 |
|  |  | Ramkumar, 2022 | Retrospective Cohort | General surgery (colectomy) | Lap & RAS | 57,710 | Race and ethnicity did not constitute a modifier for the association of rurality with receipt of MIS | 10.1001/jamanetworkopen.2022.29247 |
|  |  | Ofshteyn, 2020 | Retrospective Cohort | General surgery (proctectomy) | RAS only | 33,503 | Patients who received robotic surgery were more likely to be male, white, and privately insured. | 10.1007/s00464-019-07041-0 |
|  |  | Halloran, 2023 | Retrospective Cohort | Cardiothoracic (lobectomy) | VATs & RAS | 121,711 | Non-White patients were more likely to receive MIS lobectomy (Asian OR 5.62, p < 0.001, Black OR 1.75, p < 0.001). | 10.3390/curroncol30030213 |
|  |  | Malhotra, 2022 | Retrospective Cohort | Gynecology (excision of fallopian tubes) | Lap only | 18,725 | Hispanic women were more likely to receive open procedures as treatment for ectopic pregnancy than White women (odds ratio 1.226, p <.001). | 10.1016/j.jmig.2021.12.020 |
|  |  | Jehan, 2024 | Retrospective Cohort | Surgical oncology (distal pancreatectomy) | Lap & RAS | 13,537 | , African American patients were 30% less likely to undergo MIDP than White (OR 0.7,95% CI [0.5–0.8], p < 0.01). Similarly, Hispanic patients were 25% less likely to undergo MIDP than non-Hispanic patients (OR 0.75, 95% CI [0.6–0.9], p = 0.02). | 10.1007/s11701-023-01775-9 |
|  |  | Seldomridge, 2024 | Retrospective Cohort | Surgical oncology (pancreaticoduodenectomy for pancreatic cancer) | Lap & RAS | 16,468 | Black race negatively predicted laparoscopic use (vs white (OR 0.822; 95 % CI 0.701–0.964)) | 10.1016/j.hpb.2023.11.012 |
|  |  | Park, 2022 | Retrospective Cohort | General Surgery (gastectomy) | Lap & RAS | 41758.00 | On risk adjusted Black patients (AOR = 0.77, p = 0.024) were less likely to undergo MIS | 10.1245/s10434-021-11193-6 |
|  |  | Bachelani, 2024 | Retrospective Cohort | General Surgery (colectomy) | Lap & RAS | 100100.00 | The analysis found that a racial disparity in MIS utilization was still significant, even after adjusting for insurance status | 10.1016/j.sopen.2024.03.007 |
|  |  | Mitzman, 2022 | Retrospective Cohort | Cardiothoracic (lung resection) | VATs & RAS | 162335.00 | On multivariable analysis, African–American race, low volume, Medicaid insurance, and nonacademic setting were associated with a lower likelihood of Misurgery | 10.1002/jso.26923 |
|  |  | Logan, 2023 | Retrospective Database | Urology (Radical prostatectomy) | RAS only | 243292.00 | Patients who were non‐Hispanic Black (82.8%) or Hispanic (81.3%) had lower rates of RARP than non‐Hispanic White (84.0%) or Asian patients (87.7%, p < 0.001). | 10.1002/jso.27274 |
|  |  | Tran, 2024 | Retrospective Cohort | General Surgery (pancreatic surgery for pancreatic neoplasm) | Lap & RAS | 73137.00 | In our multivariable analysis, Black race was associated with reduced odds of MIS (AOR0.88; p = 0.02) | 10.1016/j.hpb.2024.07.403 |
|  |  | Traylor, 2020 | Retrospective Cohort | Gynecology (Hysterectomy) | Lap, RAS, Vaginal | 42945.00 | Being non-Hispanic black (OR = 0.53, 95% CI, 0.47−0.60), other or unknown race and ethnicity (OR 0.76, 95% CI, 0.52−0.85) were associated with a lower likelihood of MIS. | 10.1016/j.jmig.2020.03.014 |
| Papers that reported disparities in both outcomes and access | Single dimension of disparity analyzed | Barrington, 2022 | Retrospective Cohort | Gynecology (Hysterectomy) | Lap & RAS | 42945.00 | MIS rates were lower among Black women. Among women who underwent MIS, we observed increased read-mission risk among Black (OR: 1.21, 95% CI = 1.07, 1.37) and NativeHawaiian/Pacific Islander women (OR: 1.82, 95% CI = 1.06, 3.13) compared to White women | 10.1016/j.ygyno.2022.07.014 |
|  |  | Ko, 2021 | Retrospective Cohort | Gynecology (Hysterectomy) | Lap & Vaginal | 20,133 | Black and Hispanic women were more likely to have open surgery, Black women were more likely to experience any complication after hysterectomy (AH aOR, 1.54; 95% CI, 1.31−1.80; VH aOR,1.65; 95% CI, 1.02−2.68; LH aOR, 1.37; 95% CI, 1.13−1.66) than white women | 10.1016/j.jmig.2020.11.008 |
|  |  | AbuHasan, 2024 | Retrospective Cohort | General surgery (bariatrics - SG, bilopancreatic diversion with duodenal switch and RYGB) | Lap & RAS | 1,288,359 | Compared to White patients, Black/AA patients were more likely to undergo robotic surgery (adjusted odds ratio (aOR) 5 1.22, 95% confidence interval (CI) 5 1.21-1.24, P >.001). However, Black/AA patients were more likely to experience minor and major complications, readmissions and havepLOS compared with White patients in 2022 | 10.1016/j.soard.2024.09.002 |
|  |  | Edwards, 2023 | Retrospective Cohort | General surgery (bariatrics - RYGB) | Lap & RAS | 148,791 | All-cause mortality (OR 2.23; 95% CI: 1.16–4.29),aggregate related readmission (OR 1.39; 95% CI: 1.27–1.51), related reintervention (OR 1.36; 95% CI: 1.19–1.56), and VTE(OR 1.86; 95% CI: 1.40–2.45) were more likely in NHB patients | 10.1007/s11695-023-06541-2 |
|  |  | Matabele, 2023 | Retrospective Cohort | General surgery (bariatrics - SG and RYGB) | Lap only | 150,055 | Black race as an independent predictor of open approach to RYGB (P < 0.001) and GS (P = 0.019). Open approach to RYGB was identified as a partial mediator of the independent association between Black race and any complication, minor complications, and unplanned readmission | 10.1016/j.jss.2023.03.026 |
|  | Several dimensions of disparity analyzed | Holland, 2024 | Retrospective Cohort | General Surgery (ventral hernia repair) | Lap & RAS | 544.00 | In state patients were significantly more likely to be Black or another racial minority but had higher likelihood of undergoing MIS than out of state patients | 10.3389/jaws.2024.12946 |

Supplemental Table 3: Studies included within sex domain

| **Type of outcome reported** | **Method of disparity analysis** | **Author, year** | **Study design** | **Surgical specialty and procedure** | **Form of MIS included** | **Sample size of patients** | **Findings** | **doi** |
| --- | --- | --- | --- | --- | --- | --- | --- | --- |
| Papers that reported disparities in outcomes | Single dimension of disparity analyzed | Dugan, 2020 | Retrospective cohort | General surgery (bariatrics - RYGB and SG) | Lap only | 429664 | Male gender was an independent risk factor for RYGB and SG, major complications [2.21 vs. 1.7%,p < 0.0001 (RYGB), 1.12 vs. 0.89%, p < 0.0001 (SG)], and mortality [0.23 vs. 0.12%, p < 0.0001 (RYGB), 0.10 vs. 0.05%;p < 0.0001 (SG | 10.1007/s00464-019-07106-0 |
|  | Intersectional (sex * race) disparities analyzed | Rahimi, 2024 | Retrospective cohort | General surgery (bariatrics - SG, RYGB and duodenal switch) | Lap only | 975642 | Both females and males NHBs had higher odds of postoperative com-plications compared to NHWs in sleeve gastrectomy (Female aOR:1.31, 95% CI: [1.23–1.40]; MaleaOR:1.24, 95% CI: [1.08–1.43], P , .001) and gastric bypass (Female aOR:1.24, 95% CI: [1.16–1.33]; Male aOR:1.25, 95% CI: [1.06–1.48], P , .01) | 10.1016/j.soard.2023.12.020 |
|  | Several dimensions of disparity analyzed in one paper | Chao, 2021 | Retrospective cohort | General surgery (bariatrics - SG and RYGB) | Lap only | 605782 | On a multivariable analysis, male sex was found to be significant risk factor for postoperative VTE | 10.1016/j.soard.2021.06.022 |
| Papers that reported disparities in access | Single dimension of disparity analyzed | Stodolski, 2020 | Retrospective cohort | General surgery (endoscopic hernia repair) | Lap and RAS | 846 | The rate of endoscopic groin hernia repair was significantly lower in the female group compared to in the male cohort (30% vs. 60%, P = 0.001). | 10.1016/j.jviscsurg.2019.12.006 |
|  |  | Howard, 2023 | Retrospective cohort | General surgery (ventral and incisional hernia repair) | Lap and RAS | 5269 | Being a female was associated with a higher odds of laparoscopic/robotic repair [aOR 1.26 (95% CI 1.10-1.44)]. | 10.1007/s00464-022-09475-5 |
|  | Several dimensions of disparity analyzed in one paper |  |  |  |  |  |  |  |
|  |  | Ofshteyn, 2020 | Retrospective cohort | General surgery (colorectal surgery) | Lap and RAS | 33503 | Patients who received robotic surgery were more likely to be male, white, and privately insured. | 10.1007/s00464-019-07041-0 |
|  |  | Patel, 2022 | Retrospective cohort | General surgery (proctectomy) | Lap and RAS | 82965 | No difference was seen in female and male patients for MIS (p = 0.20) | 10.1007/s00464-021-08690-w |

Supplemental Table 4: Studies included within rurality/urbanicity domain

| **Type of outcome reported** | **Method of disparity analysis** | **Author, year** | **Study design** | **Surgical specialty and procedure** | **Form of MIS included** | **Sample size of patients** | **Findings** | **doi** |
| --- | --- | --- | --- | --- | --- | --- | --- | --- |
| Papers that reported disparities in access | Single dimension of disparity analyzed | Howell, 2024 | Retrospective cohort | General surgery (Cholecystectomy) | Lap only | 505,481 | Using urban hospitals as the reference, no difference was detected in the rate of laparoscopy | 10.1016/j.amjsurg.2024.115852 |
|  | Intersectional (race * urbanicity) disparities analyzed | Hayanga, 2024 | Retrospective cohort | Cardiothoracic (lung lobectomy) | RAS only | 13403 | Black/urban patients had significantly higher RAS rates (P < .001), and fewer open resections (P < .001). .002), compared with rural White patients (P [ .005) | 10.1016/j.athoracsur.2024.03.040 |
|  | Intersectional (race*urbanicity) | Riner, 2023 | Cross sectional | General surgery (colorectal surgery) | Both Lap & RAS | 216364 | Hispanic patients living in metropolitan areas had much higher odds of MIS [OR 1.34 (95% CI 1.11–1.61), p = 0.0020] compared with NHB [OR 1.12 (95% CI 1.01–1.24), p = 0.0308] and NHW [OR 1.08 (95% CI 1.05–1.11), p < 0.0001] patients. | 10.1245/s10434-023-13693-z |
|  | Several dimensions of disparity analyzed in one paper | Ramkumar, 2022 | Retrospective cohort | General surgery (colectomy) | Both Lap & RAS | 57710 | Residents of rural areas ( adjusted OR, 0.75 [95% CI, 0.70-0.80]) were significantly less likely to undergo MIS | 10.1001/jamanetworkopen.2022.29247 |
|  |  | Ofshteyn, 2020 | Retrospective cohort | General surgery (proctectomy) | RAS only | 33503 | Patients who received robotic surgery were more likely to live in metropolitan areas | 10.1007/s00464-019-07041-0 |
|  |  | Halloran, 2023 | Retrospective cohort | Thoracic (lung lobectomy) | VATs & RAS | 121711 | MIS was less likely in rural settings (OR 0.75, p < 0.001),and in the western region of the United States (OR 0.49, p < 0.001) | 10.3390/curroncol30030213 |
|  |  | Jehan, 2024 | Retrospective cohort | Surgical oncology (distal pancreatectomy) | Both Lap & RAS | 13537 | No difference was seen in rural-urban patients for MIS (p = 0.130) | 10.1007/s11701-023-01775-9 |
|  |  | Logan, 2023 | Retrospective cohort | Urology (radical prostatectomy) | RAS only | 243292 | Patients from rural or nonmetropolitan areas were less likely to receive RAS than those residing in metropolitan areas (78.7% rural vs 81.3% nonmetropolitan vs 84.3% metropolitan, p<0.001) | 10.1002/jso.27274 |
|  |  | Erhunmunsee 2020 | Retrospective cohort | Cardiothoracic (lung lobectomy) | RAS only | 139800 | Compared to patients in urban teaching hospitals, patients in rural hospitals were much less likely to undergo robotic versus open (AOR =0.28, P<0.01) or VATS (AOR =0.64, P<0.01) lobectomy. | 10.21037/vats.2020.02.01 |

Supplemental Table 5: Studies included within payor/insurance status domain

| **Type of outcome reported** | **Method of disparity analysis** | **Author, year** | **Study design** | **Surgical specialty and procedure** | **Form of MIS included** | **Sample size of patients** | **Findings** | **doi** |
| --- | --- | --- | --- | --- | --- | --- | --- | --- |
| Papers that reported disparities in outcomes | Single dimension of disparity analyzed | Wu, 2022 | Retrospective cohort | General surgery (sleeve gastrectomy) | Both Lap & RAS | 243 | Postoperative weight loss outcomes were similar across patients who were at safety-net vs. private hospitals. The two groups had similar percent excess weight loss (EWL) at all timepoints up to 36 months and similar rates of failure to achieve 50% EWL at 12 months | 10.1007/s11695-022-06307-2 |
|  |  | Liu, 2021 | Retrospective cohort | General surgery (sleeve gastrectomy and Roux-en-Y gastric bypass) | Lap only | 647 | In adjusted analysis, medicaid status was not associated with weight loss outcomes | 10.1097/as9.0000000000000028 |
| Papers that reported disparities in access | Intersectional (race*payor status) | Riner, 2023 | Cross sectional | General surgery (colorectal surgery) | Both Lap & RAS | 216,364 | Insurance was found to be a significant predictor for use of MIS and NHB patients were more likely to be on Medicaid and less likely to receive MIS | 10.1245/s10434-023-13693-z |
|  | Single dimension of disparity analyzed | Childers, 2023 | Cross sectional | General surgery (colorectal surgery) | Both Lap & RAS | 597,414 | On adjusted analysis, patients with Medicaid (odds ratio [OR] 0.86 [CI 0.79–0.95]) and the Uninsured (OR 0.67 [CI 0.56–0.79]) had lower odds of receiving a robotic operation than those with Private insurance in 2019. | 10.1245/s10434-023-13354-1 |
|  |  | Lee, 2023 | Retrospective cohort | General surgery (colorectal resection) | Both Lap & RAS | 7866 | Medicaid patients had lower odds of receiving MIS than private insurance patients (OR 0.85, 95% CI [0.74-0.97], p = 0.017), and the total cost was higher by $5043 USD (p < 0.001) in the Medicaid group for managing inflammatory bowel disease surgically | 10.1007/s00464-023-10400-7 |
|  |  | Dingillo, 2023 | Retrospective cohort | Cardiothoracic (lung resection) | VATs and RAS | 41439 | Medicaid expansion was associated with increased MIS utilization in unadjusted analysis (10,278/20,446 (50.3%) vs 9,953/20,993 (47.4%), p < .001) and in multivariable difference in difference analysis (ATE 0.6%, 95% CI 0.3-0.8%, P = .008). | 10.1177/00031348221138081 |
|  |  | Eguia, 2020 | Retrospective cohort | General surgery (non-emergent surgical procedure for obesity,paraesophageal hernia, gastroesophageal reflux disease, chronic diverticulitis) | Lap and Nissen fundoplication | 117241 | Following the enactment of the affordable care act, use of both laparoscopic gastric bypass (IRR 1.08; 95% CI: [1.02, 1.15]) and Nissen fundoplication(IRR 1.17; 95% CI [1.09, 1.26]) increased in Medicaid patients treated in expansion states than in those treated in non-expansion states | [10.1245/s10434-022-12663-1](https://doi.org/10.1245/s10434-022-12663-1) |
|  |  | Hrebinko, 2021 | Retrospective cohort | General surgery (colectomy) | Both Lap & RAS | 802304 | Patients treated at safety-net hospitals were less likely to receive MIS | 10.1016/j.jss.2021.03.012 |
|  |  | Branche, 2024 | Retrospective cohort | General surgery (colectomy) | Both Lap & RAS | 532640 | After adjustment, care at safety-net hospitals remained independently associated with reduced odds of minimally invasive surgery (adjusted odds ratio 0.92; 95% confidence interval 0.87e0.97) | 10.1016/j.surg.2024.03.036 |
|  |  | Dugan, 2024 | Retrospective cohort | Cardiothoracic (transhiatal esophagectomy) | RAS only | 85 | Despite Medicare patients being older and often having a broader operative history, hospital costs and reimbursements did not differ from patients with private insurance post-robotic surgery. The robotic platform appears to mitigate potential disparities in hospitalization costs and hospital reimbursement between Medicare and private insurance | 10.1007/s11701-023-01762-0 |
|  |  | Gilja, 2024 | Retrospective cohort | ENT (transoral robotic surgery) | RAS only | 19810 | In terms of surgical approach, patients treated at higher safety-net burden facilities received RAS less frequently than patients treated at lower safety-net burden facilities (HBH: 13.1% vs. MBH: 25.4% vs. LBH: 35.2%,p < 0.001) | 10.1002/lary.31131 |
|  |  | Badrinathan, 2023 | Retrospective cohort | Cardiothoracic ( esophagectomy) | Both Lap & RAS | 8820 | Minimally invasive esophagectomy was more common in expansion states (30.37%vs 23.88%, P < .001). A multivariable difference-in-differences analysis, however, suggested no effect in MIS rate due to Medicaid expansion. | 10.1177/26345161231178351 |
|  |  | Ng, 2022 | Retrospective cohort | Gynecology (gynecologic cancer resection) | Both Lap & RAS | 462529 | MIS was associated with less financial toxicity for uninsured, public insured and Black patients than Open | 10.1016/j.ygyno.2022.05.017 |
|  | Multiple dimensions of disparity analyzed | Holland, 2024 | Retrospective cohort | 544 | Both Lap & RAS | General surgery (ventral hernia repair) | In state patients were more likely to undergo MIS (OVHR: 45.6%vs. 81.5%, laparoscopic: 38.2% vs. 14.1%, robotic: 16.2% vs. 4.4%; p < 0.001) when compared to out of state referrals | 10.3389/jaws.2024.12946 |
|  |  | Patel, 2022 | Retrospective cohort | 82,965 | Both Lap & RAS | General surgery (colectomy) | Medicaid and uninsured patients had decreased odds of MIS use during hospitalization compared to private insurance (OR 0.751,p < 0.0001 and OR 0.629, p < 0.0001 respectively). | 10.1007/s00464-021-08690-w |
|  |  | Tracy, 2021 | Retrospective cohort | 559 | Both Lap & RAS | General surgery (Abdominal wall herniorrhaphy) | The most important variables identified by our model to predict modality of surgery were job growth (for RAS), insurance type (for Lap), and no high school diploma (for Open surgery) | 10.1007/s00464-020-07860-6 |
|  |  | Anastasio, 2024 | Retrospective cohort | 21,837 | Both Lap & RAS | Gynecology (Hysterectomy) | Patients with private insurance were most likely to undergo MIS (74.7%); those with Medicaid were least likely to undergoMIS (55.7%). Patients with Medicare were most likely to undergo an LND(68.6%); those with Medicaid were least likely to undergo an LND | 10.1016/j.ajog.2024.09.002 |
|  |  | Ofshteyn, 2020 | Retrospective cohort | 33,503 | RAS only | General surgery (proctectomy) | Patients who received robotic surgery were more likely to be male, white, and privately insured. | 10.1007/s00464-019-07041-0 |
|  |  | Halloran, 2023 | Retrospective cohort | 121,711 | VATs and RAS | Cardiothoracic (Lobectomy) | Multivariable analysis showed that thoracic surgeon provider type was less likely with medicaid patients | 10.3390/curroncol30030213 |
|  |  | Malhotra, 2022 | Retrospective cohort | 18,725 | Lap only | Gynecology (Excision of fallopian tubes) | Women with private insurance were more likely to receive open procedures than women who used self-pay for treatment (odds ratio 0.809, p <.001). | 10.1016/j.jmig.2021.12.020 |
|  |  | Jehan, 2024 | Retrospective cohort | 13,537 | Both Lap & RAS | Surgical oncology (Distal pancreatectomy) | Compared to Medicare/private insured patients, uninsured patients were 50% less likely to undergo MIS (OR 0.5, 95% CI [0.4–0.7], p < 0.01). | 10.1007/s11701-023-01775-9 |
|  |  | Seldomridge, 2024 | Retrospective cohort | 16,468 | Both Lap & RAS | Surgical oncology (pancreaticoduodecetomy) | Predictors of RAS included Medicare/other government insurance (vs uninsured or Medicaid (OR 1.660;95 % CI 1.123–2.454)) and private insurance (vs uninsured or Medicaid (OR 1.597; 95 % CI1.090–2.340)) | 10.1016/j.hpb.2023.11.012 |
|  |  | Mitzman, 2022 | Retrospective cohort | 162335 | VATs and RAS | Cardiothoracic (lung resection) | On multivariable analysis, African–American race, low volume, Medicaid insurance, and nonacademic setting were associated with a lower likelihood of MIS surgery | 10.1002/jso.26923 |
|  |  | Kosber, 2022 | Retrospective cohort | 9088 | RAS only | Urology (reteral reimplantation) | There was a significantly decreased odds among older patients (OR = 0.98, p < 0.001), those with Medicaid (OR = 0.5, p = 0.02) for robotic rather than open reteral reimplantation | 10.1089/end.2021.0415 |
|  |  | Bachelani 2024 | Retrospective Cohort | 100100.00 | Both Lap & RAS | General surgery (colectomy) | Patients with private insurance were more likely to have an MIS operation. There was also a racial disparity in MIS utilization, even after adjusting for insurance status | 10.1016/j.sopen.2024.03.007 |
|  |  | Tran 2024 | Retrospective Cohort | 73137.00 | Both Lap & RAS | General surgery (proctectomy) | In our multivariable analysis, private insurance coverage (AOR 1.30; p = 0.05) were associated with increased odds. | 10.1016/j.hpb.2024.07.403 |
|  |  | Logan, 2023 | Retrospective cohort | 243292 | RAS only | Urology (radical prostatectomy) | Medicaid or uninsured patients were less likely to receive RARP (75.5%) compared to patients with Medicare or private insurance (84.4%, p < 0.001). Medicaid or uninsured status was associated with decreased odds of RARP in adjusted multivariable analysis (OR 0.61, 95% CI 0.49–0.76). | 10.1002/jso.27274 |
|  |  | Erhunmunsee, 2020 | Retrospective cohort | 139800 | RAS only | Cardiothoracic (lung lobectomy) | Patients who were low-income, on medicaid and uninsured patients were less likely to go through RAS than Open and VATs | 10.21037/vats.2020.02.01 |

Supplemental Table 6: Studies included within socioeconomic status/income domain

| **Type of outcome reported** | **Method of disparity analysis** | **Author, year** | **Study design** | **Surgical specialty and procedure** | **Form of MIS included** | **Sample size of patients** | **Findings** | **doi** |
| --- | --- | --- | --- | --- | --- | --- | --- | --- |
| Papers that reported disparities in outcomes | Single dimension of disparity analyzed | Jermihov, 2022 | Retrospective cohort | Cardiothoracic (lung lobectomy) | RAS only | 447 | Lower SES was significantly and positively associated with postoperative complications (odds ratio (OR)=1.98, p=0.039). | 10.7759/cureus.26201 |
|  |  | Johns, 2023 | Retrospective cohort | General surgery (Roux-en-Y gastric bypass and sleeve gastrectomy) | Lap only | 583 | Low tier DCI patients twice as likely to have >15% weight recurrence (OR 2.0, CI 1.41-2.84) | 10.1007/s00464-023-10158-y |
| Papers that reported disparities in access | Intersectional (race*SES) | Hayanga, 2024 | Retrospective cohort | Cardiothoracic (lung lobectomy) | RAS only | 13403 | Low-income Black/urban patients had higher RAS (P [ .002), and fewer open resections (P < .001) compared with rural White patients (P [ .005) | 10.1016/j.athoracsur.2024.03.040 |
|  | Intersectional (race*SES) | Riner, 2023 | Cross sectional | General surgery (colorectal surgery) | Both Lap and RAS | 216364 | When racial/ethnic groups were stratified, the odds of undergoing MIS approach were even lower for Non Hispanic Black patients in the lowest income quartile | 10.1245/s10434-023-13693-z |
|  | Several dimensions of disparity analyzed in one paper | Tracy, 2021 | Retrospective cohort | General surgery (abdominal wall herniorrhaphy) | Both Lap and RAS | 559 | DCI and rates of poverty were both significant predictors of open surgery (p=0.01) compared to MIS | 10.1007/s00464-020-07860-6 |
|  |  | Anatasio, 2024 | Cross sectional | Gynecology (Hysterectomy) | Both Lap and RAS | 21837 | Compared to bottom quartile, patients with higher income quartiles had a higher proportion of MIS | 10.1007/s00464-020-07860-6  10.1016/j.ajog.2024.09.002 |
|  |  | Halloran, 2023 | Retrospective cohort | Cardiothoracic (lung lobectomy) | VATs and RAS | 121711 | Factors associated with a decreased likelihood of patients receiving care from a thoracic provider for lung lobectomy included non-White race, lower socioeconomic status, and receipt of care in a rural hospital setting. | 10.3390/curroncol30030213 |
|  |  | Ramkumar, 2022 | Retrospective cohort | General surgery (colectomy) | Both Lap and RAS | 57710 | Higher Area Deprivation Index was independently associated with less MIS (OR 0.93 [95% CI, 0.92-0.94]). | 10.1001/jamanetworkopen.2022.29247 |
|  |  | Ofshteyn, 2020 | Retrospective cohort | General surgery (proctectomy) | RAS only | 33503 | Patients who received RAS were more likely to be from ZIPs with higher high school graduation rates. Living less than 25 miles away from the hospital was associated with a smaller likelihood of robotic approach (OR 0.79,95% CI 0.72–0.86, p < 0.001) | 10.1007/s00464-019-07041-0 |
|  |  | Malhotra, 2022 | Retrospective cohort | Gynecology (Excision of fallopian tubes) | Lap only | 18725 | Women of lower median income status, <$60 000, were more likely to receive open procedures than women of the fourth quartile income group. Black women predominantly made up the first quartile income group. | 10.1016/j.jmig.2021.12.020 |
|  |  | Jehan, 2024 | Retrospective cohort | Surgical oncology (distal pancreatectomy) | Both Lap and RAS | 13537 | Based on the medium household income, compared to patients in the fourth quartile, patients in all other quartiles were less likely to undergo MIS | 10.1007/s11701-023-01775-9 |
|  |  | Seldomridge, 2024 | Retrospective cohort | Surgical oncology (pancreaticoduodenectomy) | Both Lap and RAS | 16468 | Patients who underwent Lap were more likely to have an income in the top quartile (41.41 % vs 38.54 % vs 37.42 %) and less likely to have an income in the bottom quartile (14.66 % vs 15.83 % vs 17.09 %; p = 0.040) compared to open and RAS, respectively | 10.1016/j.hpb.2023.11.012 |
|  |  | Park, 2022 | Retrospective cohort | General surgery (gastectomy) | Both Lap and RAS | 41758 | On risk adjusted analysis, black patients (AOR = 0.77, p = 0.024) and patients with income below 25th percentile (AOR = 0.80, p = 0.018) were less likely to undergo MIS | 10.1245/s10434-021-11193-6 |
|  |  | Erhunmunsee, 2020 | Retrospective cohort | Cardiothoracic (lung lobectomy) | RAS only | 139800 | Patients who were low-income were less likely to go through RAS than Open and VATs | 10.21037/vats.2020.02.01 |
|  |  | Sakowitz, 2024 | Retrospective cohort | Cardiothoracic (lung lobectomy) | VATs and RAS | 217762 | Decreased odds for MIS was reported for lower quartiles of median neighborhood income (51st-75th percentile adjusted odds ratio, 0.92, 95% CI, 0.89-0.94; 26th-50th percentile adjusted odds ra-tio, 0.86, CI, 0.83-0.89; 0-25th percentile adjusted odds ratio, 0.78, CI, 0.75-0.81;reference: 76th-100th percentile income) | 10.1016/j.jtcvs.2023.12.008 |
